# Genetic Heritage of the Balto-Slavic Speaking Populations: A Synthesis of Autosomal, Mitochondrial and Y-Chromosomal Data

**DOI:** 10.1371/journal.pone.0135820

**Published:** 2015-09-02

**Authors:** Alena Kushniarevich, Olga Utevska, Marina Chuhryaeva, Anastasia Agdzhoyan, Khadizhat Dibirova, Ingrida Uktveryte, Märt Möls, Lejla Mulahasanovic, Andrey Pshenichnov, Svetlana Frolova, Andrey Shanko, Ene Metspalu, Maere Reidla, Kristiina Tambets, Erika Tamm, Sergey Koshel, Valery Zaporozhchenko, Lubov Atramentova, Vaidutis Kučinskas, Oleg Davydenko, Olga Goncharova, Irina Evseeva, Michail Churnosov, Elvira Pocheshchova, Bayazit Yunusbayev, Elza Khusnutdinova, Damir Marjanović, Pavao Rudan, Siiri Rootsi, Nick Yankovsky, Phillip Endicott, Alexei Kassian, Anna Dybo, The Genographic Consortium, Chris Tyler-Smith, Elena Balanovska, Mait Metspalu, Toomas Kivisild, Richard Villems, Oleg Balanovsky

**Affiliations:** 1 Evolutionary Biology Group, Estonian Biocentre, Tartu, Estonia; 2 Institute of Genetics and Cytology, National Academy of Sciences of Belarus, Minsk, Belarus; 3 Department of Genetics and Cytology, Karazin Kharkiv National University, Kharkіv, Ukraine; 4 Vavilov Institute of General Genetics, Russian Academy of Sciences, Moscow, Russia; 5 Research Centre for Medical Genetics, Russian Academy of Sciences, Moscow, Russia; 6 Department of Human and Medical Genetics, Faculty of Medicine, Vilnius University, Vilnius, Lithuania; 7 Institute of Mathematical Statistics, University of Tartu, Tartu, Estonia; 8 Center for Genomics and Transcriptomics (CeGaT GmbH), Tübingen, Deutschland; 9 Faculty of Pharmacy, University of Sarajevo, Sarajevo, Bosnia and Herzegovina; 10 Department of Evolutionary Biology, Institute of Molecular and Cell Biology, University of Tartu, Tartu, Estonia; 11 Faculty of Geography, Lomonosov Moscow State University, Moscow, Russia; 12 Belgorod State University, Belgorod, Russia; 13 Kuban State Medical University, Krasnodar, Russia; 14 Northern State Medical University, Arkhangel, Russia; 15 Institute of History, National Academy of Sciences of Belarus, Minsk, Belarus; 16 Institute of Biochemistry and Genetics, Ufa Research Centre, RAS, Ufa, Bashkortostan, Russia; 17 Department of Genetics and Fundamental Medicine of Bashkir State University, Ufa, Bashkortostan, Russia; 18 International Burch University, Sarajevo, Bosnia and Herzegovina; 19 Institute for Anthropological Research, Zagreb, Croatia; 20 Musée de l'Homme, Paris, France; 21 Institute of Linguistics, Russian Academy of Sciences, Moscow, Russia; 22 School for Advanced Studies in the Humanities, Russian Presidential Academy of National Economy and Public Administration, Moscow, Russia; 23 The Wellcome Trust Sanger Institute, Hinxton, Cambs, United Kingdom; 24 Department of Archaeology and Anthropology, University of Cambridge, Cambridge, United Kingdom; 25 Estonian Academy of Sciences, Tallinn, Estonia; Universitat Pompeu Fabra, SPAIN

## Abstract

The Slavic branch of the Balto-Slavic sub-family of Indo-European languages underwent rapid divergence as a result of the spatial expansion of its speakers from Central-East Europe, in early medieval times. This expansion–mainly to East Europe and the northern Balkans–resulted in the incorporation of genetic components from numerous autochthonous populations into the Slavic gene pools. Here, we characterize genetic variation in all extant ethnic groups speaking Balto-Slavic languages by analyzing mitochondrial DNA (n = 6,876), Y-chromosomes (n = 6,079) and genome-wide SNP profiles (n = 296), within the context of other European populations. We also reassess the phylogeny of Slavic languages within the Balto-Slavic branch of Indo-European. We find that genetic distances among Balto-Slavic populations, based on autosomal and Y-chromosomal loci, show a high correlation (0.9) both with each other and with geography, but a slightly lower correlation (0.7) with mitochondrial DNA and linguistic affiliation. The data suggest that genetic diversity of the present-day Slavs was predominantly shaped *in situ*, and we detect two different substrata: ‘central-east European’ for West and East Slavs, and ‘south-east European’ for South Slavs. A pattern of distribution of segments identical by descent between groups of East-West and South Slavs suggests shared ancestry or a modest gene flow between those two groups, which might derive from the historic spread of Slavic people.

## Introduction

Balto-Slavic speakers comprise around one-third of present-day Europeans and occupy nearly a half of the European subcontinent. There is a near consensus among linguists that the Baltic and Slavic languages stem from a common root, Proto-Balto-Slavic, which separated from other Indo-European languages around 4,500–7,000 years before present (YBP) [1–8] and whose origin is mapped to Central Europe [8]. The Balto-Slavic node was recognized already in the pioneer Indo-European tree by [9]. The split between Baltic and Slavic branches has been dated to around 3,500–2,500 YBP [6–8], whereas further diversification of the Slavic languages probably occurred much later, around 1,700–1,300 YBP according to [6–8,10–12]. The phenomenon of the “Slavicization” of Europe–dispersion of the Slavic languages–was discussed in early studies [13–15].

Although there is no single archaeological signature for their spread, historical records suggest that a major Slavic expansion across Europe took place approximately 1,400–1,000 YBP [16–19]; reviewed recently in [20]. The Slavic expansion in Eastern Europe affected areas previously occupied by Baltic, Finno-Ugric and Turkic speaking populations; in Central-West Europe groups speaking Germanic languages; and in the Balkans populations of diverse linguistic affiliation [10,11,18,19,21].

The question of to what extent this recent cultural transformation within Europe affected its genetic landscape has been the subject of numerous studies. Uniparental genetic markers, mitochondrial DNA (mtDNA) and the non-recombining part of the Y-chromosome (NRY), indicate that the genetic composition of Slavs does not differ significantly from that of their neighboring non-Slavic populations [22–34]. In addition, age estimates for major paternal and maternal lineages of East-Central Europe point to an expansion that pre-dates the historic spread of Slavs. For example, whilst the geographic distribution of NRY haplogroups (hg) I-P37 and R1a-Z282 overlaps with the area occupied by the present-day Slavs, coalescent times suggest that the current diversity within these hgs existed prior to the Slavic expansion [29,35]. Similarly, the phylogeography of mtDNA hgs that are more frequent in West and East Slavs–such as H5a1, U4a2, U5a2a, U5a2b1 –suggests continuity within East-Central Europe for at least two thousand years [28,36–38]. While these genetic components predated the Slavic expansion, a recent study on the distribution of genomic segments identical by descent (IBD) among different European populations revealed a high number of shared segments among East Europeans that can be dated to around 1,000–2,000 YBP [39]. Similarly, multi-directional admixture events among East Europeans (both Slavic and non-Slavic), dated to around 1,000–1,600 YBP, were inferred in [40]. Both patterns were interpreted as genetic signals for the movements of people during a period that includes the proposed time-frame for the Slavic expansion. Until now, however, no genome-scale study focusing on Balto-Slavic populations has been available and only a small number of groups have been included in genome-wide SNP scans of genetic diversity in Europe [41–48].

Here, our aim is to contribute to a comprehensive understanding of patrilineal, matrilineal and autosomal genetic variation in the Balto-Slavic-speaking peoples. The Balto-Slavic “case” allows us to test correlations across these three genetic systems in well-established linguistic and geographic space, and to address questions about the genetic history of the carriers of this large linguistic subfamily within the neighboring non-Balto-Slavic Indo-European, Finno-Ugric, North Caucasian and Turkic speakers. To do so, we analyze 6,876 mtDNAs, 6,079 NRYs and 296 whole genome SNP profiles representing all extant Balto-Slavic populations, of which 917, 2,392 and 70, respectively, are reported here for the first time. We complement our genetic study with linguistic evidence, in particular by refining the phylogeny of the extant Slavic languages.

## Results

### Genetic structuring of Balto-Slavic populations

**Fig 1:**
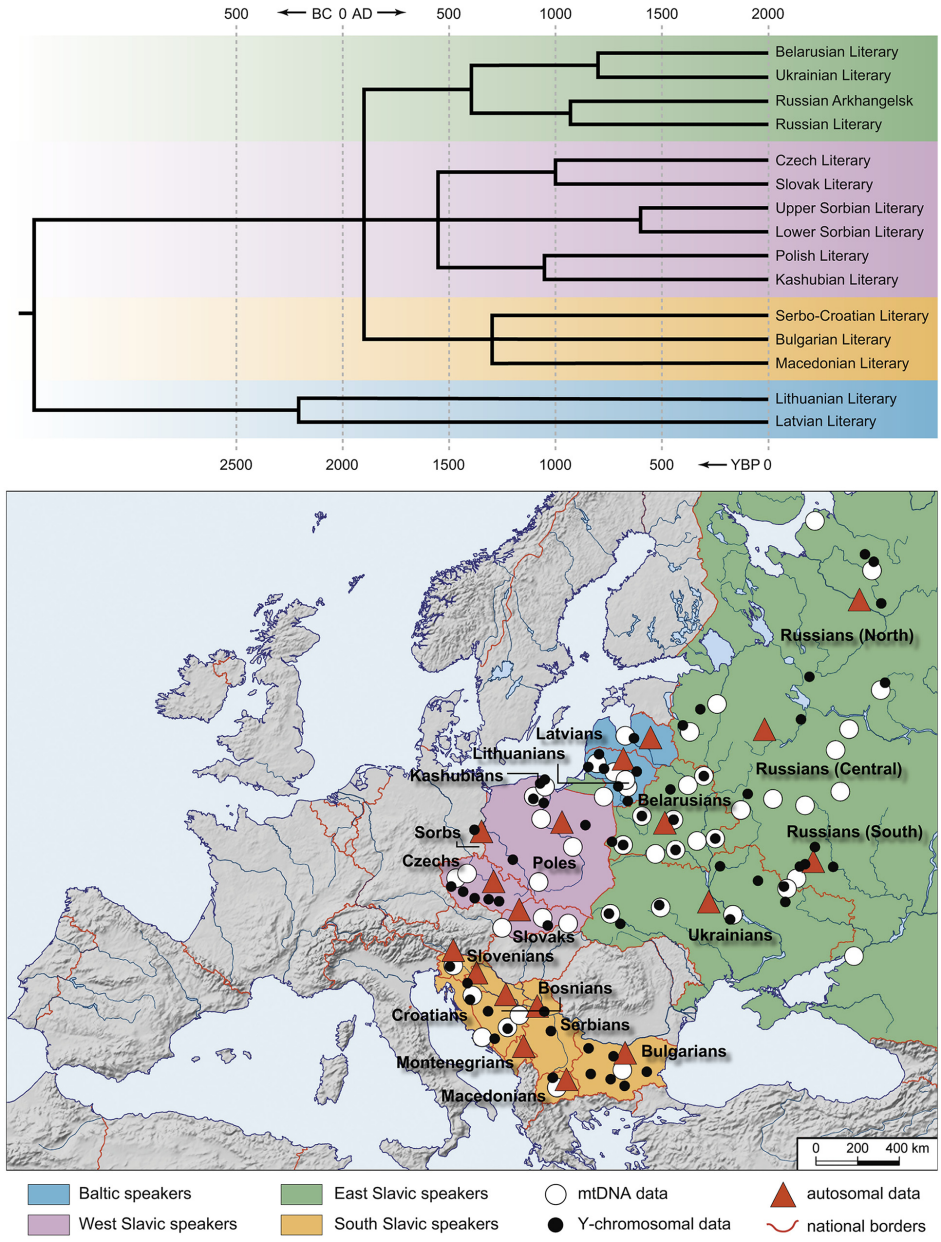
The Balto-Slavic populations analyzed in this study and the tree of Balto-Slavic languages. The map (lower panel) shows the geographical distribution of Balto-Slavic populations (colored areas) within Europe. The symbols on the map represent the geographic location of the populations genotyped. The map was created in the GeneGeo software as described previously [68,75]. A manually constructed consensus phylogenetic tree of the Balto-Slavic languages (upper panel) is based on the StarlingNJ, NJ, BioNJ, UPGMA, Bayesian MCMC, Unweighted Maximum Parsimony methods. Ternary nodes resulting from neighboring binary nodes were joined together if the temporal distance between them was ≤ 300 years. StarlingNJ dates are proposed (S2 File).

**Fig 2:**
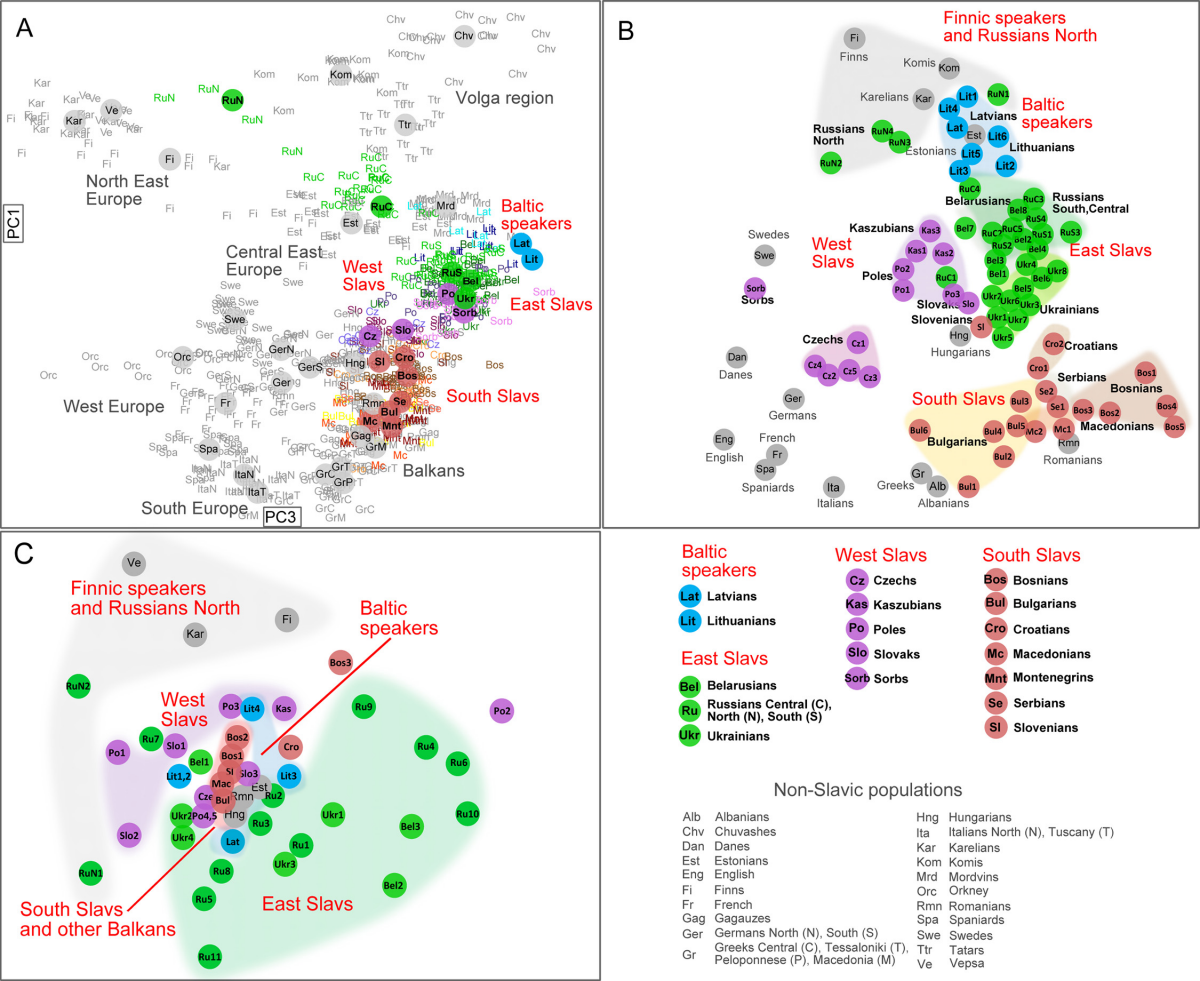
Genetic structure of the Balto-Slavic populations within a European context according to the three genetic systems. a) PC1vsPC3 plot based on autosomal SNPs (PC1 = 0.53; PC3 = 0.26); b) MDS based on NRY data (stress = 0.13); c) MDS based on mtDNA data (stress = 0.20). We focus on PC1*vs*PC3 because PC2 (S1 Fig) whilst differentiating the Volga region populations from the rest of Europeans had a low efficiency in detecting differences among the Balto-Slavic populations–the primary focus of this work.

The genetic structuring of Balto-Slavic populations (Fig 1) in a European context is shown in three plots, representing autosomal PC1*vs*PC3, NRY and mtDNA MDS analyses, respectively (Fig 2A, 2B and 2C). In the autosomal-and NRY-based plots, most Balto-Slavic populations are dispersed along the north-south axis of their geographic origin (Fig 2A and 2B). In their Y-chromosomal and autosomal variation, East Slavs–Russians from central-southern regions, Belarusians and Ukrainians– form a cluster on their own, though these populations do not overlap entirely with each other (Fig 2A and 2B). This group is characterized by low mean values of population pairwise genetic distances (D_Nei_ = 0.125 for NRY; F_ST_ = 0.0008 for autosomal data) (Tables A,B in S1 File). In contrast, Russians from the northern region of the European part of Russia are differentiated from the rest of the East Slavs, and on genetic plots lie in the vicinity of their Finnic-speaking geographic neighbors. Accordingly, the average genetic distances between North Russians and the rest of East Slavic populations are high: D_Nei_ = 0.584; F_ST_ = 0.0081) (Tables A,B in S1 File). Compared to the East Slavs, the West Slavs are more differentiated. In particular, Czechs (Fig 2A and 2B) and to a lesser extent also Slovaks (Fig 2A), are shifted towards Germans and other West Europeans, whereas Poles either overlap or lie close to East Slavs. Likewise, population pairwise genetic distances are as twice as high for West Slavs as for East Slavs (D_Nei_ = 0.241 for NRY; F_ST_ = 0.0014) (Tables A,B in S1 File). Notably, genetic distances remain low after adding Poles to the Belarusians, Ukrainians and Russians from the central-southern regions (D_Nei_ = 0.144 for NRY; F_ST_ = 0.0006 for autosomal data), indicating thereby an extended geographic area with low genetic differentiation among the majority of Slavic speakers across Central-East Europe.

Most South Slavs are separated from the rest of the Balto-Slavic populations and form a sparse group of populations with internal differentiation into western (Slovenians, Croatians and Bosnians) and eastern (Macedonians and Bulgarians) regions of the Balkan Peninsula with Serbians placed in-between (Fig 2A and 2B). The mean population pairwise genetic distances for South Slavs (D_Nei_ = 0.239 for NRY; F_ST_ = 0.0009 for autosomal data) (Tables A,B in S1 File) are comparable or higher to the ones for East Slavs despite the smaller region within the Balkan Peninsula that they occupy. Furthermore, Slovenians lie close to the non-Slavic-speaking Hungarians, whereas eastern South Slavs group is located together with non-Slavic-speaking but geographically neighboring Romanians and, to some extent, with Greeks.

Both extant Baltic-speaking populations, Latvians and Lithuanians, lie in the vicinity of Finno-Ugric-speaking Estonians according to their Y-chromosome diversity (Fig 2B), whilst in their autosomal variation they are slightly shifted towards the group of East Slavic speakers (Fig 2A). Also, one finds Volga-Finnic Mordvins close to the two Baltic-speaking populations (Fig 2A), potentially reflecting historic evidence that the Baltic-speaking tribes’ spread zone formerly reached more eastward parts of the East European Plain [49,50].

The patterns of genetic structure of the Balto-Slavic populations agree particularly between autosomal and NRY data. However, the maternal gene pool of the Balto-Slavic populations, although less structured possibly due to somewhat lower phylogenetic resolution of the dataset (Fig 2C, Tables C, D in S1 File), bears some features similar to those of autosomal and NRY ones such as the differentiation of North Russians and the overlap between East Slavs (Fig 2A, 2B and 2C). In contrast to mtDNA and even to autosomes, the NRY variation often reveals its fine structuring within the Balto-Slavic patrilineal gene pool (Fig 2B, see also Table E in S1 File).

### Ancestral components of the Balto-Slavic gene pool

**Fig 3:**
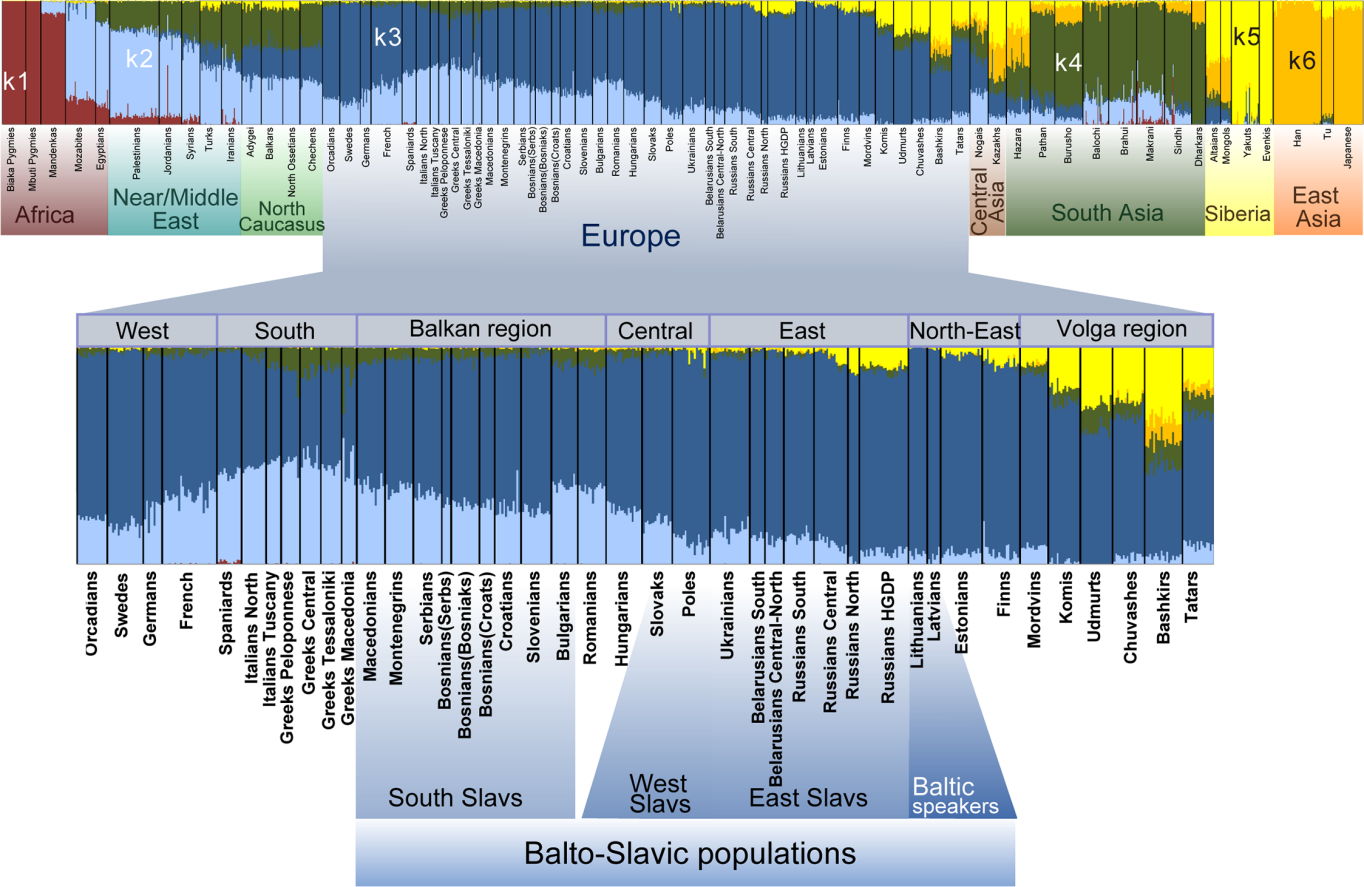
ADMIXTURE plot (k = 6). Ancestry proportions of 1,194 individuals as revealed by ADMIXTURE.

Using the clustering algorithm implemented in ADMIXTURE [51], we modeled ancestral genetic components in Balto-Slavic populations. Assuming six ancestral populations (K = 6) (see S1 Text: Methods for choosing a best K), Balto-Slavic speakers bear membership almost exclusively from two ancestral components: the *dark blue* (k3) and the *light blue* (k2), albeit in different proportions (Fig 3). k3 is omnipresent throughout European populations and decreases from north-eastern Europeans southwards. Thus, k3 peaks in Baltic speakers and prevails in East Slavs (80–95%) and decreases notably in South Slavs (55–70%). In contrast, k2 is abundant around the Mediterranean and in the Caucasus region and decreases among Europeans when moving northward. Accordingly, it makes up nearly 30% of ancestral proportions in South Slavs, decreases to around 20% in West and East Slavs and drops to around 5% in North Russians and Baltic speakers (Fig 3). The further division of the two major components (k3 and k2) in the Balto-Slavic populations at higher values of K indicates more complex structuring of genomes of South Slavs as compared to West and East Slavs (S2 Fig).

As far as minor ancestral components are concerned, only West and East Slavs, and, predominantly North Russians, bear the ‘Siberian/Volga-region’ component (k5, *lemon yellow*) (Fig 3). It is noteworthy that the k6 component, predominant among Han Chinese and abundant in Mongols and Altaians, is virtually absent in Russians, suggesting that the “East Eurasian” share in North and Central Russian ancestry is due to admixture with North-Central Siberians, rather than with South Siberia/Mongols (Fig 3, S2 Fig).

### Distribution of segments identical by descent among Balto-Slavic speakers and surrounding populations

To analyze further the patterns of gene flow among the Balto-Slavic populations and their non-Slavic neighbors as well as to explore the genetic heritage of the suggested Slavic migration from Central-East to the Balkan region of Europe, we focused on the pairwise sharing of IBD segments [39,52] and applied the *fIBD* algorithm [53]. We created two groups of Slavs–East-West Slavs (1) and South Slavs (2)–and seven additional groups of populations representing the geographic context for present-day Slavs (S3 Fig; Table F in S1 File). As a measure of IBD sharing, we used an average number of IBD segments per pair of individuals (which we refer to as ibd-statistic). We calculated the ibd-statistic for the two groups of Slavic speakers, and compared it to the ibd-statistic for each of the groups of Slavs and their respective non-Slavic neighboring groups of populations (S3 Fig and Table F in S1 File, S1 Text: Methods for detailed description of the analysis).

**Fig 4:**
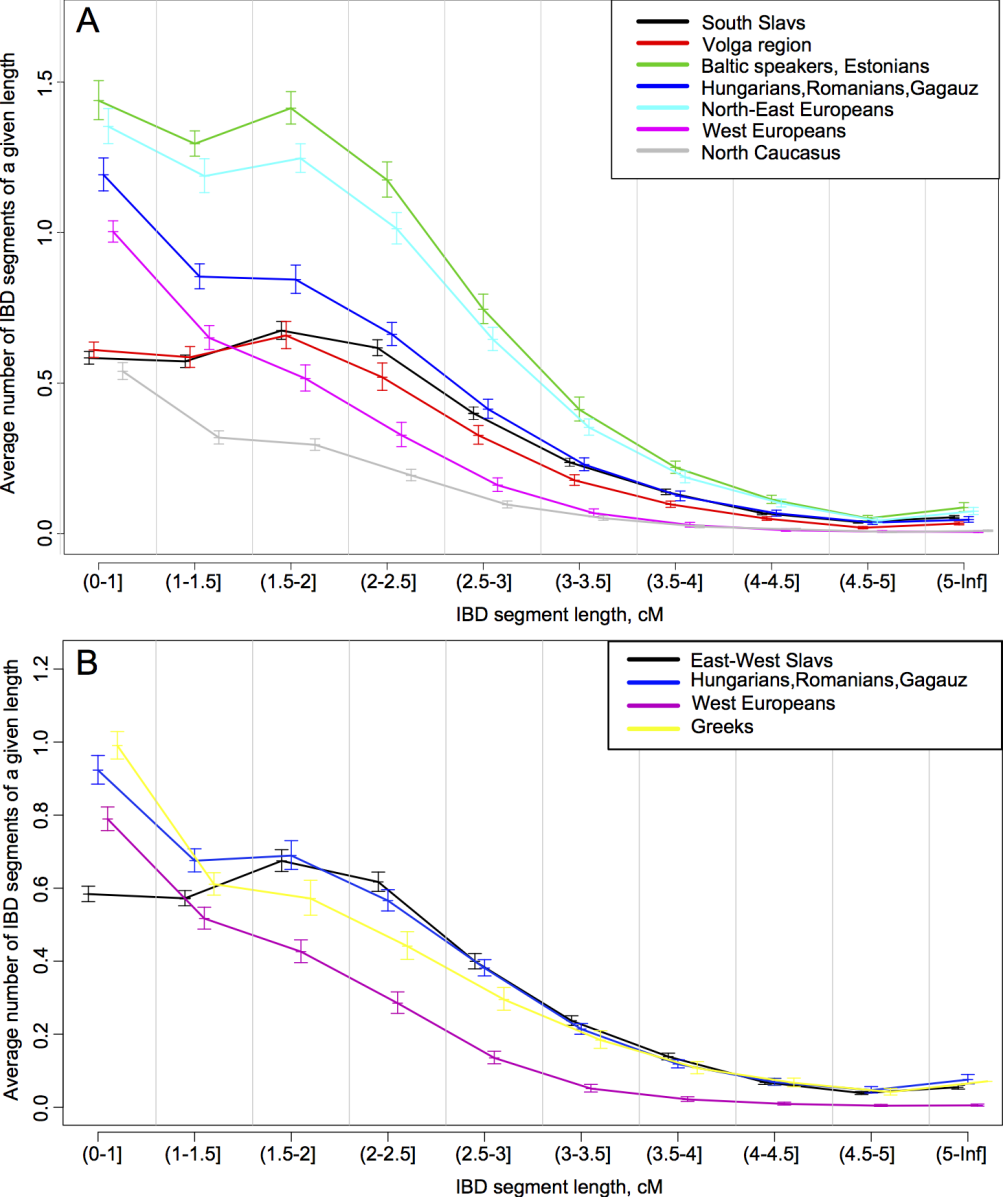
Distribution of the average number of IBD segments between groups of East-West Slavs (a), South Slavs (b), and their respective geographic neighbors. The x-axis indicates ten classes of IBD segment length (in cM); the y-axis indicates the average number of shared IBD segments per pair of individuals within each length class.

IBD analysis (Fig 4A, Table G in S1 File) reveals three patterns of IBD sharing relevant to the group of East-West Slavs in a European context. Firstly, the ibd-statistics for East-West Slavs and South Slavs (within-Slavic IBD sharing) are significantly higher than those for East-West Slavs and populations of the Volga region, West Europeans and North Caucasians (p<<0.01) (Fig 4A, Table G in S1 File). Secondly, however, this level of within-Slavic IBD sharing is lower than among East-West Slavs and populations from north-east Europe (i.e. Baltic speakers/Estonians; Karelians/Vepsa/Russians North): East-West Slavs share twice as many IBD segments with north-east Europeans as with South Slavs (p<<0.01) (Table G in S1 File). Note that exclusion of the North Russian population from the group of north-east Europeans did not lead to a significant drop in the IBD sharing between East-West Slavs and north-east Europeans (S4 Fig). Finally, the ibd-statistics for East-West Slavs and South Slavs do not differ (p = 0.08–0.8) from that of East-West Slavs and the ‘inter-Slavic’ group of populations, i.e. Hungarians, Romanians and Gagauz (Table G in S1 File, Fig 4A).

South Slavs in their turn share a similar number of IBD segments with East-West Slavs and with the ‘inter-Slavic’ Romanian, Hungarian and Gagauz populations (Fig 4B; Table G in S1 File). Notably, South Slavs share significantly fewer IBD segments for length classes 1.5–3 cM with their immediate geographic neighbors in south–Greeks–than with the group of East-West Slavs (Fig 4B).

Altogether, the analysis of IBD segment distributions revealed even patterns of IBD sharing among East-West Slavs–‘inter-Slavic’ populations (Hungarians, Romanians and Gagauz)–and South Slavs, i.e. across an area of assumed historic movements of people including Slavs.

### Lexicostatistical reconstruction of the Balto-Slavic languages

We applied a lexicostatistical approach to refine the phylogeny of the extant Balto-Slavic languages [6,7,54], focusing here particularly on the Slavic sub-branch topology and temporal estimates (for lexicostatistical dataset and methodology see S2 File, Figs A-M in S2 File, Tables A-C in S3 File; S1 Dataset). The initial division of Proto-Slavic remains unresolved: a ternary split into West, East and South dated to around 1900 YBP is suggested in the consensus phylogenetic tree (Fig 1 upper panel, Fig G in S2 File; see Figs B-F in S2 File for Proto-Slavic split discrepancies between different phylogenetic methods). Further diversification of the Slavic languages took place around 1300–1500 YBP, followed by shaping of the individual languages 1000–500 YBP. Our reconstruction suggests the existence of several intermediate clades–Ukrainian/Belarusian within East Slavic, Czech/Slovak and Polish/Kashubian within West Slavic–whereas a ternary structure is suggested for Serbo-Croatian, Bulgarian and Macedonian within South Slavic (Fig 1, Figs B-G in S2 File). Modern Slovenian, due to its vocabulary exhibiting a significant level of mixture with West and South Slavic languages, was excluded from the lexicostatistical analysis (for details see S2 File: The case of the Slovenian language, Figs H-M in S2 File).

### Partitioning the genetic variation according to the linguistic variation

Analysis of molecular variance (AMOVA) partitions the overall genetic diversity in a group of populations into fractions according to hierarchical levels of population structure. We analyzed the distribution of the NRY diversity among three levels of the linguistic tree of Balto-Slavic languages (see S1 Text, S5 Fig). The NRY diversity at the lowest level1 of the population structure–among local populations speaking the same language–varies from almost 0 within Czechs and Macedonians to 0.05 within North Russians, being on average about 0.01 (Table H in S1 File). The genetic differentiation among ethnic populations belonging to the same linguistic branch (level2) is around 0.03, and variation among branches (level3) of Balto-Slavic languages increases to 0.06 (Table H in S1 File).

### Correlation between genetic, geographic and linguistic distances of Balto-Slavic populations

**Fig 5:**
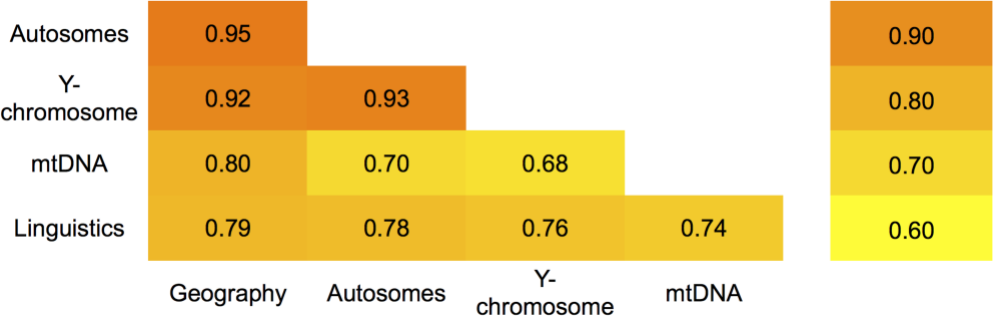
Correlations between matrices of genetic, geographic and linguistic distances among Balto-Slavic populations.

A Mantel test was applied to compare the roles which geography and language have played in shaping the genetic variation of the Balto-Slavic populations (Fig 5, Tables I,J in S1 File). The test was performed independently for the three genetic systems, with all three exhibiting a very high correlation with geography (0.80–0.95) and slightly lower (0.74–0.78) correlation with linguistics (Table J in S1 File). Because the linguistic pattern itself is highly correlated with geography (Fig 5), partial correlations were considered to distinguish between the direct and indirect influences of geography on the two other systems. The correlations with linguistics became much lower whilst all three genetic systems maintained high correlations with geography (Table J in S1 File).

## Discussion

### Two major genetic substrata are embedded in the gene pools of Slavs

The results of our study have shown the close genetic proximity of the majority of West and East Slavic populations inhabiting the geographic area from Poland in the west, to the Volga River in the East (Fig 2A and 2B, Tables A,B in S1 File). Some mtDNA haplotypes of hgs H5, H6, U4a were more frequent in the genomes of West and East Slavic speakers, providing thereby further evidence for the matrilineal unity of West and East Slavs [28,36] as well as continuity of mtDNA diversity in the territory of modern Poland for at least two millennia [38].

In contrast to this apparent genetic homogeneity of the majority of West and East Slavs, the gene pool of South Slavs, who are confined to the geographically smaller Balkan Peninsula, differs substantially and shows internal differentiation, as testified by their NRY and autosomal variation (Fig 2A and 2B; Fig 3, Tables A,B in S1 File). Consequently, we suggest that there is a “central-east European” genetic substratum in West and East Slavs, exemplified by NRY hgs R1a and the k3 ancestry component, and a “south-east European” one, featuring NRY hgs I2a and E plus the k2 ancestry component for South Slavs (Fig 2A and 2B, Fig 3, Table K in S1 File; Tables A,B in S1 File). Notably, the “south-east European” component does not extend to the whole Balkan Peninsula, as South Slavs are differentiated from Greek sub-populations except Macedonian Greeks (Fig 2A, Fig 4B) [55].

The importance of these substrata in shaping the genetic diversity of the present-day Slavs is evident from the observed lower IBD relatedness between the combined group of East-West Slavs and South Slavs than with north-east Europeans, including Baltic speakers (Fig 4A). The latter reside within the East European Plain and presumably represent the “central-east European” pre-Slavic substratum (Fig 4A, Table G in S1 File). AMOVA results also support the substrata prevalence, because genetic variation among Slavic branches (which assimilated different substratum populations) strongly exceeds intra-branch variation (Table H in S1 File). The influence of geography in shaping the Slavic genetic heritage (Fig 5, Table J in S1 File) led to the same conclusion, because if substratum importance is the major factor shaping the genetic relationships among present-day Slavic-speaking populations, these will not reflect the relationship among expanding Slavic languages, but should instead reflect the relationships between pre-Slavic populations, which can be approximated by geographical distances between them.

### Demographic mechanisms shaping the gene pool of Slavic speakers

Most West and East Slavs of Central-East Europe form genetically a compact group of populations that, as a general rule, differ from their western (Germanic-speaking) and eastern (Finno-Ugric-speaking) neighbors (Fig 2A and 2B; Fig 4A and 4B). However, so-called ‘contact’ zones of this group with non-Slavic peoples are characterized by various patterns of genetic clines or sharp genetic borders [27,32,56–58]. For example, there is a pronounced genetic proximity between Czechs and their immediate Germanic neighbors in the west (Fig 2A and 2B, Fig 3) [27,58] that could be attributed to the pre-Slavic gene pool formation of Central-East Europeans. In contrast, a clear genetic border exists nowadays between Poles and their immediate western neighbors Germans, and even between a West-Slavic-speaking minority–Sorbs–and their German host population (Fig 2B, Tables A,B in S1 File) [43,59]. It has been suggested, that this genetic boundary predates massive resettlements of people after World War II, and could have been shaped during medieval migrations of Germanic and Slavic peoples in the Vistula and Oder River basins [60]. In the north-east, a largely autochthonous (pre-Slavic) component is detected in the gene pool of Russians from northern regions of the European part of Russia (Fig 2A, 2B and 2C, Fig 3), which agrees with previous anthropological [61,62] and genetic [32,45,56,63] studies and supports substantial admixture of expanding Slavs with indigenous populations and, perhaps, language shift in the latter.

Taken together, several mechanisms including cultural assimilation of the autochthonous populations by expanding Slavs while maintaining the pre-Slavic genetic boundaries, and *in situ* gene pool shaping, are needed to explain the genetic patterns observed on the eastern, north-eastern and western margins of the current ‘Slavic area’ within Central-East Europe.

The presence of two distinct genetic substrata in the genomes of East-West and South Slavs would imply cultural assimilation of indigenous populations by bearers of Slavic languages as a major mechanism of the spread of Slavic languages to the Balkan Peninsula. Yet, it is worthwhile to add here evidence from the analysis of IBD segments: the majority of Slavs from Central-East Europe (West and East) share as many IBD segments with the South Slavs in the Balkan Peninsula as they share with non-Slavic populations residing nowadays between Slavs (Fig 4A and 4B; Table G in S1 File). This even mode of IBD sharing might suggest shared ancestry/gene flow across the wide area and physical boundaries such as the Carpathian Mountains, including the present-day Finno-Ugric-speaking Hungarians, Romance-speaking Romanians and Turkic-speaking Gagauz. A slight peak at 2–3 cM in the distribution of shared IBD segments between East-West and South Slavs (Fig 4A and 4B) might hint at shared “Slavonic-time” ancestry, but this question requires further investigation.

Expansion of Slavic languages took place in an area already occupied by speakers of the Baltic languages [49,50]. Despite significant linguistic divergence between extant East Baltic and Slavic languages (Fig 1) [7], Baltic populations are genetically the closest to East Slavs (Fig 2A and 2B, Table K in S1 File) [45,64–66] and here we found that they bear the highest number of shared IBD segments with the combined group of East-West Slavs (Fig 4, Table G in S1 File). The presence of a substantial “Baltic substratum” in the genomes of extant Slavs within East Europe might in part explain their genetic closeness to each other and difference from some neighboring non-Slavic groups.

### A synthesis

Comparing genetic and linguistic reconstructions with geography has a long tradition in human population genetics [67]. Here, we have studied the autosomal, NRY and mtDNA diversity of all Balto-Slavic populations in the context of their linguistic variation and geography. A remarkable agreement between these five systems was found: correlation coefficients range from 0.68 to near the maximum (0.95). This agreement between datasets from different systems supports the reliability of the results and in most cases, when drawing a conclusion, we could find one supported by the majority of the systems analyzed. In particular, we found that autosomal and NRY compositions and geographic affiliations of the Balto-Slavic populations form a triad, all variables of which are very similar to each other.

Combining all lines of evidence, we suggest that the major part of the within-Balto-Slavic genetic variation can be primarily attributed to the assimilation of the pre-existing regional genetic components, which differed for West, East and South Slavic-speaking peoples as we know them today.

## Materials and Methods

### Ethics Statement

The DNA samples analysed in the study were collected after having obtained written informed consent. The procedure has been approved by Ethics Committees of the appropriate Institutions, including the Research Ethics Committee of the University of Tartu (UT REC) (no 225/T-9) and the Ethics Committee of the Research Centre for Medical Genetics, Russian Academy of Sciences.

### Datasets

Three datasets NRY, mtDNA and autosomal SNP representing populations speaking Balto-Slavic languages were assembled. *The NRY data* comprises 6,079 samples, including 1,254 reported here for the first time and 1,138 samples updated from previous work (Table L in S1 File). *The mtDNA data* include 6,876 samples, 917 are reported here for the first time (Table C in S1 File). *The autosomal SNP data* include 1,297 worldwide individuals including 70 reported here for the first time (Table M in S1 File); this dataset encompasses in total 296 samples representing Balto-Slavic populations. S1 Text: Datasets provides extended information on dataset assemblage. All samples reported here for the first time were collected after informed consent was obtained from each participant.

### Genotyping

40 binary NRY markers were genotyped using the TaqMan (Applied Biosystems) technology as described [68]. MtDNA analyses included HVS1 sequencing and genotyping of coding region SNPs defining mtDNA hgs [69] (mtDNA tree Build 15 (30 Sep 2012). The autosomal SNP genotypes were generated with the Illumina 660K array and combined with published data (Table M in S1 File). S1 Text: Methods provides details about the autosomal SNP pre-processing performed before all analyses.

### MDS, PCA and ADMIXTURE

MDS analysis based on genetic distances [70] was performed for the NRY and mtDNA datasets (Tables C, K, N in S1 File). PCA was performed for the autosomal dataset using the *smartpca* program of the EIGENSOFT package [71]; sets of Illumina-Affymetrix cross-platform SNPs (around 57k of LD-pruned SNPs), encompassing available Balto-Slavic populations, were used. Genomic ancestry components in Balto-Slavic speakers in the context of worldwide populations were inferred with ADMIXTURE [51]; sets of only Illumina cross-platform SNPs (around 200k shared LD-pruned SNPs between the 610K, 650K and 660K arrays) were used (Table M in S1 File). See S1 Text: Methods for choosing the value of K which best models the ancestry components in our dataset.

### Analysis of pairwise segments IBD

We aimed to compare the level of IBD relatedness between the combined group of East-West Slavs (group1) *vs* South Slavs (group2) (i.e. IBD relatedness within Slavs) to the IBD relatedness between each group of Slavs *vs* their respective neighboring groups of mostly non-Slavic populaitons (Table F in S1 File lists populations in each group, S3 Fig shows schematically the geographic location of each population groups). To this end we: a) calculated an average number of IBD segments per pair of individuals (ibd-statistic) between the group of East-West Slavs (group1) and South Slavs (group2), i.e. within-Slavic IBD sharing, and between each Slavic group and their respective geographic neighbors; b) compared the within-Slavs ibd-statistic with the ibd-statistics for each Slavic group and groups 3–9. The fast IBD (*fIBD*) algorithm [53] implemented in BEAGLE (http://faculty.washington.edu/browning/beagle/beagle.html) was used to detect pairwise IBD segments. Sets of Illumina-only cross-platform SNPs (around 500k shared SNPs between the 610K, 650K and 660K arrays) were used in the analysis. See S1 Text: Methods for detailed information about the experimental design and statistical approach applied.

### AMOVA and Mantel tests

AMOVA (implemented in the Arlequin 3.11) was applied to estimate genetic differentiation when Balto-Slavic populations were grouped according to the three hierarchical levels of the tree of Balto-Slavic languages (S1 Text: Methods, Table H in S1 File, S5 Fig). Mantel tests were performed in Arlequin 3.11 [72] to calculate the coefficients of the pairwise and partial correlations between matrices of genetic (mtDNA, NRY and whole genome SNP), linguistic and geographic distances (Table I in S1 File). S1 Text: Methods provides additional details for Mantel tests analysis.

### Lexicostatistical reconstruction of Balto-Slavic languages

20 wordlists of extant Balto-Slavic languages were used to reconstruct their phylogeny. The consensus tree (Fig 1, Fig G in S2 File) was drawn manually based on the set of trees produced by different phylogenetic methods. The method implying individual relative index of stability for each Swadesh item [73,74] was used for the node dating. S2 File, Figs A-C in S2 File, and Tables A,B in S3 File contain detailed information about lexicostatistical reconstruction of the Balto-Slavic languages.

## Supporting Information

S1 Dataset (zip-archive).
bslav.dbf, bslav.var, bslav.inf, lexical dataset in STARLING format (multistate matrix with synonyms allowed). This dataset exported in MS EXCEL format is available as Table A in S3 File.bslav.nex, the same dataset as a binary matrix in NEXUS format.*.tre, some of the discussed trees in NEWICK format;NEXUS files for NeighborNet networks.
(ZIP)

S1 Fig PC1*vs*PC2 plot based on whole genome SNP data (PC1 = 0.53; PC2 = 0.34). (PDF)

S2 Fig ADMIXTURE plot (k2-k20) (A). Box and whiskers plot of the cross validation (CV) indexes of all runs of the ADMIXTURE analysis (B). Log-likelihood (LL) scores of all runs (C). Variation in LL scores in the fractions (5%, 10%, 20% shown in dark green, middle green and light green, respectively) of runs that reached the highest LLs) (D). (PDF)

S3 Fig Schematic representation of groups of populations used in the IBD analysis. Populations within each group are listed in Table F in S1 File. Source of the Europe contour map: http://www.conceptdraw.com/How-To-Guide/geo-map-europe.
(PDF)

S4 Fig Distribution of the average number of IBD segments between group of East-West Slavs and their geographic neighbors. Russians from Northern region of European part of Russia are considered separately from the group of north-east Europeans. The x-axis indicates ten classes of IBD segment length (in cM); the y-axis indicates the average number of shared IBD segments per pair of individuals within each length class.
(PDF)

S5 Fig Hierarchical levels of genetic variation used in AMOVA. (PDF)

S1 File Table A in S1 File. Matrix of pairwise Nei distances (D_Nei_) between Balto-Slavic populations based on Y-chromosome data. Table B in S1 File. Matrix of mean population pairwise F_ST_ for Balto-Slavic populations calculated from autosomal SNP data. Table C in S1 File. Frequencies of the mtDNA haplogroups in Balto-Slavic and some other European populations. Table D in S1 File. Matrix of pairwise Nei distances (D_Nei_) between Balto-Slavic populations based on mtDNA data. Table E in S1 File. Predicting the country affiliation for 53 Balto-Slavic populations from their Y-chromosomal composition. Table F in S1 File. Groups of populations used in IBD analysis. Table G in S1 File. Summary statistics of IBD analysis. Table H in S1 File. Analysis of molecular variance (AMOVA) in Balto-Slavic populations. Table I in S1 File. Matrices of geographic (a), lexicostatistical (b) and genetic (c,d,e) distances between Balto-Slavic populations used in Mantel Tests. Table J in S1 File. Results for Mantel tests on genetic, lexicostatistical and geographic distances. Table K in S1 File. Frequencies of the NRY haplogroups in Balto-Slavic populations. Table L in S1 File. Frequencies of NRY haplogroups in 29 Balto-Slavic populations presented here for the first time. Table M in S1 File. Populations used in whole-genome SNP analyses. Table N in S1 File. Frequencies of the NRY haplogroups in non-Balto-Slavic populations of Europe. (XLSX)

S2 File (Linguistics: Datasets; Methods; Results). Fig A in S2 File. Geographical distribution of extant Slavic and East Baltic languages and dialects used in the study. Map was prepared by Yuri Koryakov. Fig B in S2 File. Dated phylogenetic tree of the Balto-Slavic lects produced by the StarlingNJ method from the multistate matrix (binary nodes only). Bootstrap values are shown near the nodes (not shown for stable nodes with bootstrap value ≥ 95%). Fig C in S2 File. Phylogenetic tree of the Balto-Slavic lects produced by the NJ method from the binary matrix in the SplitsTree4 software. Bootstrap values are shown near the nodes (not shown for stable nodes with bootstrap value ≥ 95%). Branch length reflects the relative rate of cognate replacement as suggested by SplitsTree4. The BioNJ method yields the same topology. Fig D in S2 File. Phylogenetic tree of the Balto-Slavic lects produced by the UPGMA method from the binary matrix in the SplitsTree4 software. Bootstrap values are shown near the nodes (not shown for stable nodes with bootstrap value ≥ 95%). Branch length reflects the relative rate of cognate replacement as suggested by SplitsTree4. Fig E in S2 File. Consensus phylogenetic tree of the Balto-Slavic lects produced by the Bayesian MCMC method from the binary matrix in the MrBayes software. Bayesian posterior probabilities are shown near the nodes (not shown for stable nodes with P ≥ 0.95). Branch length reflects the relative rate of cognate replacement as suggested by MrBayes. Fig F in S2 File. Optimal phylogenetic tree of the Balto-Slavic lects produced by the UMP method from the binary matrix in the TNT software. Bootstrap values are shown near the nodes (not shown for stable nodes with bootstrap value ≥ 95%). Branch length reflects the relative rate of cognate replacement as suggested by TNT. Fig G in S2 File. Manually constructed consensus phylogenetic tree of the Balto-Slavic lects based on the StarlingNJ, NJ, BioNJ, UPGMA, Bayesian MCMC, UMP methods. Ternary nodes result from neighboring binary nodes, joined together, if the temporal distance between them ≤ 300 years. The gray ellipses additionally mark two joined nodes, which cover binary branchings that differ depending on the method. Probability values are shown in the following sequence: NJ/Bayesian MCMC/UMP (“x” means that P ≥ 0.95 in an individual method; not shown for nodes with P ≥ 0.95 in all methods). StarlingNJ dates are proposed. Fig H in S2 File. NeighborNet network of the Balto-Slavic lects (without Slovenian) + German. Produced in the SplitsTree4 software; bootstrap values are shown near the nodes (not shown for stable nodes with bootstrap value ≥ 95%). Fig I in S2 File. NeighborNet network of the Balto-Slavic lects (without Slovenian) + Demotic Greek. Produced in the SplitsTree4 software; bootstrap values are shown near the nodes (not shown for stable nodes with bootstrap value ≥ 95%). Fig J in S2 File. NeighborNet network of the Balto-Slavic lects (without Slovenian) + German + Demotic Greek. Produced in the SplitsTree4 software; bootstrap values are shown near the nodes (not shown for stable nodes with bootstrap value ≥ 95%). Fig K in S2 File. NeighborNet network of the Balto-Slavic lects (with Slovenian) + German. Produced in the SplitsTree4 software; bootstrap values are shown near the nodes (not shown for stable nodes with bootstrap value ≥ 95%). Fig L in S2 File. NeighborNet network of the Balto-Slavic lects (with Slovenian) + Demotic Greek. Produced in the SplitsTree4 software; bootstrap values are shown near the nodes (not shown for stable nodes with bootstrap value ≥ 95%). Fig M in S2 File. NeighborNet network of the Balto-Slavic lects (with Slovenian) + German + Demotic Greek. Produced in the SplitsTree4 software; bootstrap values are shown near the nodes (not shown for stable nodes with bootstrap value ≥ 95%).
(PDF)

S3 File Table A in S3 File. Lexical dataset (multistate matrix with synonyms allowed). Table B in S3 File. Reverse distance matrix generated from the multistate matrix (Table A in S3 File) in the Starling software. Table C in S3 File. Distance matrix, generated from the binary matrix (bslav.nex (deposited in S1 Dataset)) in the SplitsTree4 software. (XLSX)

S1 Text (Genetics: Datasets, Methods). (DOCX)
