# Supplementary material for: Genetic Heritage of the Balto-Slavic Speaking Populations: A Synthesis of Autosomal, Mitochondrial and Y-Chromosomal Data
Source: PLoS One. 2015 Sep 2;10(9):e0135820. doi: 10.1371/journal.pone.0135820 (PMC4558026; doi:10.1371/journal.pone.0135820)
Supplement: S1 Fig — (PDF) [file pone.0135820.s002.pdf]

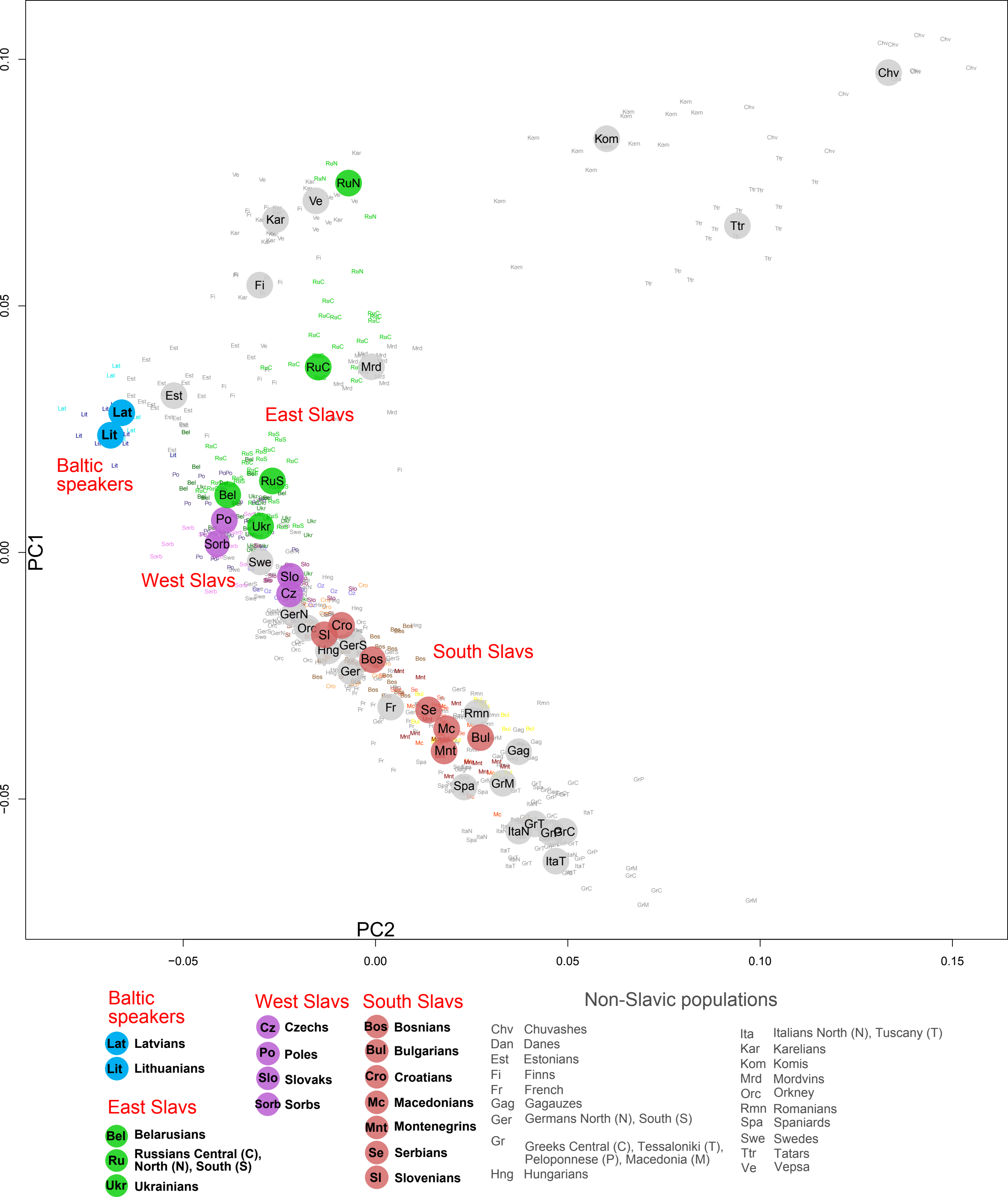

0.10
0.05
0.00
−0.05
PC1
−0.05
0.00
0.05
0.10
0.15
PC2
Chv
Kom
Ttr
RuN
Ve
Kar
Fi
RuC
Mrd
Est
Lat
Lit
East Slavs
Baltic speakers
RuS
Bel
Po
Ukr
Sorb
Swe
West Slavs
Slo
Cz
GerN
Orc
Sl
Cro
Hng
GerS
Bos
Ger
South Slavs
Fr
Se
Rmn
Mc
Bul
Mnt
Gag
GrM
Spa
ItaN
GrT
GrP
GrC
ItaT
Baltic speakers
Lat Latvians
Lit Lithuanians
East Slavs
Bel Belarusians
Ru Russians Central (C), North (N), South (S)
Ukr Ukrainians
West Slavs
Cz Czechs
Po Poles
Slo Slovaks
Sorb Sorbs
South Slavs
Bos Bosnians
Bul Bulgarians
Cro Croatians
Mc Macedonians
Mnt Montenegrins
Se Serbians
Sl Slovenians
Non-Slavic populations
Chv Chuvashes
Dan Danes
Est Estonians
Fi Finns
Fr French
Gag Gagauzes
Ger Germans North (N), South (S)
Gr Greeks Central (C), Tessaloniki (T), Peloponnese (P), Macedonia (M)
Hng Hungarians
Ita Italians North (N), Tuscany (T)
Kar Karelians
Kom Komis
Mrd Mordvins
Orc Orkney
Rmn Romanians
Spa Spaniards
Swe Swedes
Ttr Tatars
Ve Vepsa
