# Supplementary material for: Genetic Heritage of the Balto-Slavic Speaking Populations: A Synthesis of Autosomal, Mitochondrial and Y-Chromosomal Data
Source: PLoS One. 2015 Sep 2;10(9):e0135820. doi: 10.1371/journal.pone.0135820 (PMC4558026; doi:10.1371/journal.pone.0135820)
Supplement: S3 Fig — Populations within each group are listed in Table F in S1 File. Source of the Europe contour map: http://www.conceptdraw.com/How-To-Guide/geo-map-europe. (PDF) [file pone.0135820.s004.pdf]

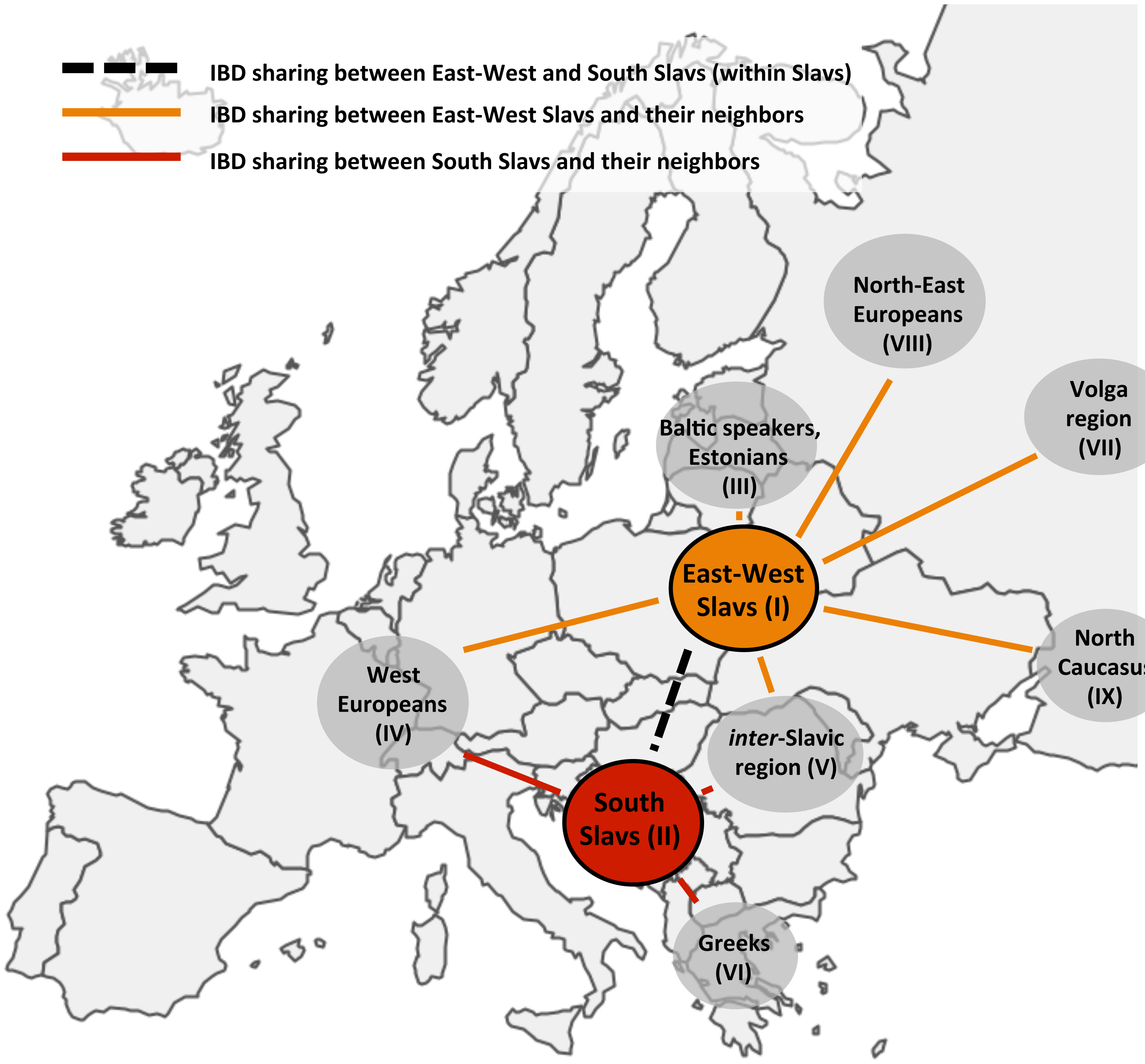
IBD sharing between East-West and South Slavs (within Slavs)
IBD sharing between East-West Slavs and their neighbors
IBD sharing between South Slavs and their neighbors
North-East Europeans (VIII)
Volga region (VII)
Baltic speakers, Estonians (III)
East-West Slavs (I)
North Caucasus (IX)
West Europeans (IV)
inter-Slavic region (V)
South Slavs (II)
Greeks (VI)
