# Supplementary figures and images for: Genetic Heritage of the Balto-Slavic Speaking Populations: A Synthesis of Autosomal, Mitochondrial and Y-Chromosomal Data
Source: PLoS One. 2015 Sep 2;10(9):e0135820. doi: 10.1371/journal.pone.0135820 (PMC4558026; doi:10.1371/journal.pone.0135820)

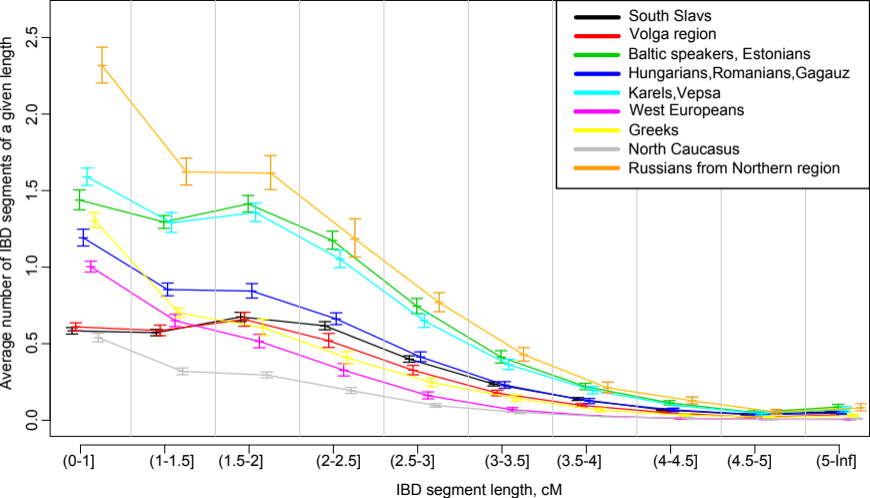

Supplement: S4 Fig — Russians from Northern region of European part of Russia are considered separately from the group of north-east Europeans. The x-axis indicates ten classes of IBD segment length (in cM); the y-axis indicates the average number of shared IBD segments per pair of individuals within each length class. (PDF) [file pone.0135820.s005.pdf]
