# Supplementary material for: Genetic Heritage of the Balto-Slavic Speaking Populations: A Synthesis of Autosomal, Mitochondrial and Y-Chromosomal Data
Source: PLoS One. 2015 Sep 2;10(9):e0135820. doi: 10.1371/journal.pone.0135820 (PMC4558026; doi:10.1371/journal.pone.0135820)
Supplement: S5 Fig — (PDF) [file pone.0135820.s006.pdf]

S5 Fig. Hierarchical levels of genetic variation in AMOVA

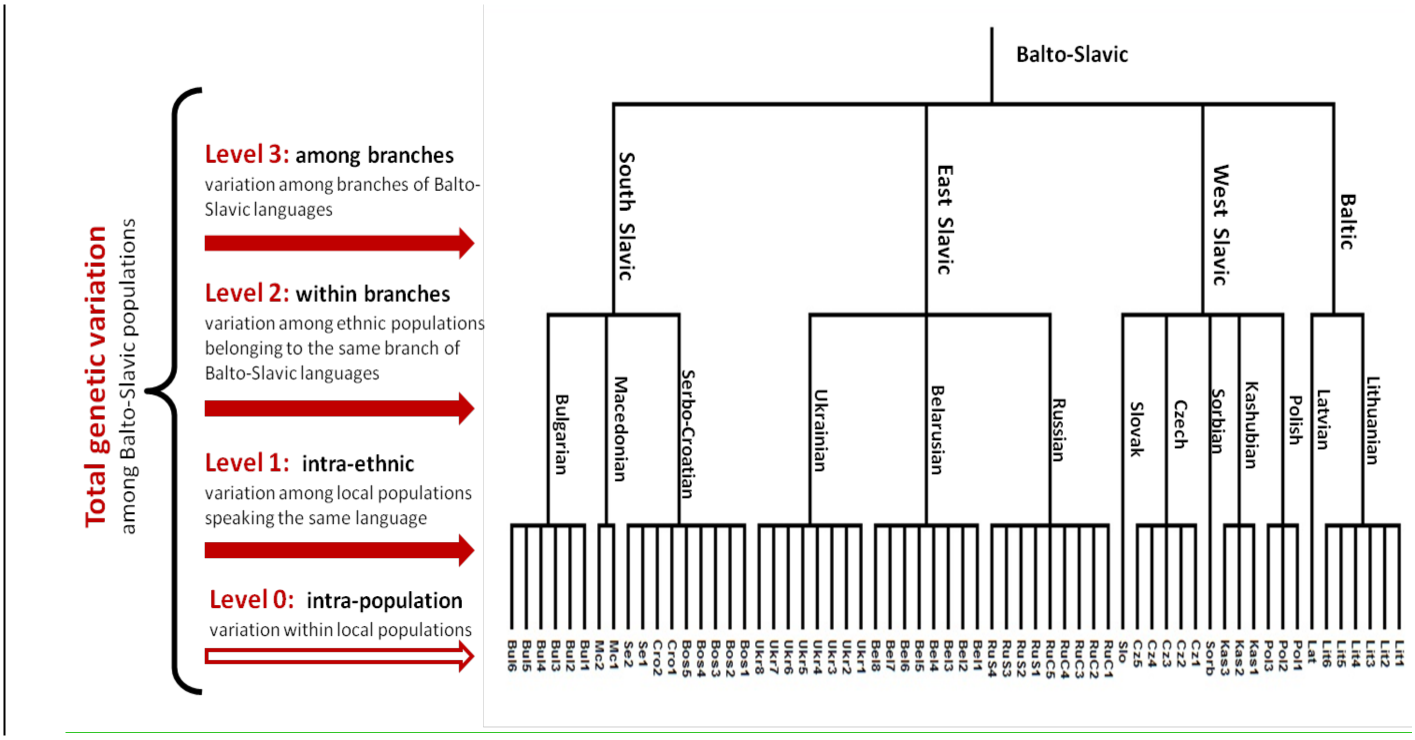

NOTES:

1. The scheme shows the principle of populations grouping for AMOVA. This tree demonstrates grouping according to the linguistic classification (Fig. 1).
2. At the level 1 populations, speaking the same language, were grouped together. Only languages with more than one studied local population were analyzed; thus, intra-ethnic variation within Latvians, Sorbs and Slovaks was not estimated. Northern Russians were not included in analysis because of their significant genetic peculiarity.
3. At the levels 2 and 3 we used mean haplogroup frequencies in all groups (including languages represented by single studied populations).
4. Although split into Baltic and Slavic languages occurred much earlier that further ternary split of Slavic languages, we have not introduced the fourth hierarchical level to avoid over complexity of the analysis.
5. For population abbreviations see Table K in S1 File.
