# Supplementary material for: Genetic Heritage of the Balto-Slavic Speaking Populations: A Synthesis of Autosomal, Mitochondrial and Y-Chromosomal Data
Source: PLoS One. 2015 Sep 2;10(9):e0135820. doi: 10.1371/journal.pone.0135820 (PMC4558026; doi:10.1371/journal.pone.0135820)
Supplement: S2 File — Fig A in S2 File. Geographical distribution of extant Slavic and East Baltic languages and dialects used in the study. Map was prepared by Yuri Koryakov. Fig B in S2 File. Dated phylogenetic tree of the Balto-Slavic lects produced by the StarlingNJ method from the multistate matrix (binary nodes only). Bootstrap values are shown near the nodes (not shown for stable nodes with bootstrap value ≥ 95%). Fig C in S2 File. Phylogenetic tree of the Balto-Slavic lects produced by the NJ method from the binary matrix in the SplitsTree4 software. Bootstrap values are shown near the nodes (not shown for stable nodes with bootstrap value ≥ 95%). Branch length reflects the relative rate of cognate replacement as suggested by SplitsTree4. The BioNJ method yields the same topology. Fig D in S2 File. Phylogenetic tree of the Balto-Slavic lects produced by the UPGMA method from the binary matrix in the SplitsTree4 software. Bootstrap values are shown near the nodes (not shown for stable nodes with bootstrap value ≥ 95%). Branch length reflects the relative rate of cognate replacement as suggested by SplitsTree4. Fig E in S2 File. Consensus phylogenetic tree of the Balto-Slavic lects produced by the Bayesian MCMC method from the binary matrix in the MrBayes software. Bayesian posterior probabilities are shown near the nodes (not shown for stable nodes with P ≥ 0.95). Branch length reflects the relative rate of cognate replacement as suggested by MrBayes. Fig F in S2 File. Optimal phylogenetic tree of the Balto-Slavic lects produced by the UMP method from the binary matrix in the TNT software. Bootstrap values are shown near the nodes (not shown for stable nodes with bootstrap value ≥ 95%). Branch length reflects the relative rate of cognate replacement as suggested by TNT. Fig G in S2 File. Manually constructed consensus phylogenetic tree of the Balto-Slavic lects based on the StarlingNJ, NJ, BioNJ, UPGMA, Bayesian MCMC, UMP methods. Ternary nodes result from neighboring binary nodes [file pone.0135820.s008.pdf]

Alena Kushniarevich et al. Genetic heritage of the Balto-Slavic speaking populations: a synthesis of autosomal, mitochondrial and Y-chromosomal data.

Supplementary Information 2: LINGUISTIC DATA

by Alexei Kassian & Anna Dybo

S2: 1. Lexicographic sources & dataset compilation criteria

Our Balto-Slavic lexical dataset comprises 110-item wordlists of 20 modern lects (i.e., languages and dialects). Besides the lexicographic sources listed below, we used unpublished wordlists, previously prepared for the *Tower of Babel* project (S. Starostin 1998–2005) by Vasiliy Chernov (Yandex company). Additionally, we consulted the comparative study by Mikhail Saenko (2013).

Slavic

East Slavic

- Russian Literary: compiled by V. Chernov, revised by A. Dybo. Sources:
  - Evgenyeva, A. P. (ed.). 1999. *Slovar' russkogo yazyka v 4-kh tomakh*. Moscow: Russkij yazyk. Vol. 1–4.
  - Vasmer, M. 1950–1958. *Russisches etymologisches Wörterbuch*. Heidelberg: C. Winter.
- Russian Bolshoe Davydovskoe (Vladimir-Volga dialect group, Большое Давыдовское village, Gavrilovo-Posadsky district, Ivanovo province, Russia): compiled by S. Nikolaev, revised by A. Dybo. Sources:
  - Field records by S. Nikolaev, 1990s.
- Russian Smekhnovo (Pskov dialect group, Смехново village, Andreapolsky district, Tver province, Russia): compiled by S. Nikolaev, revised by A. Dybo. Sources:
  - Field records by S. Nikolaev, 1990s.
- Russian Arkhangelsk (Northern Russian dialect group, Arkhangelsk province, Russia): compiled by A. Dybo. Sources:
  - *Arkhangel'skij oblastnoj slovar'*. Moscow: Nauka, 1980–2013. Vol. 1–15.
  - Unpublished database of *Arkhangel'skij oblastnoj slovar'* containing data collected in the expeditions of the Faculty of Philology of Moscow State University (1956–2013); the work on the database is headed by I. Kachinskaya

- Ukrainian Literary: compiled by V. Chernov, revised by A. Dybo & A. Kassian. Sources:
    - Bilodid, I. et al. (ed.). 1970–1980. *Slovnik ukraïns'koï movi u 11 tomakh*. Kiyiv: Naukova dumka. Vol. 1–11.
    - Golovaschuk, S. et al. (ed.). 1969. *Russko-ukrainskij slovar'*. Kiyiv: Naukova dumka. Vol. 1–3.
    - Melnichuk, O. S. 1982. *Etimologichnij slovnik ukraïns'koï movi u semi tomakh*. Kiyiv: Naukova dumka. Vol. 1–6.
- Ukrainian Tisiv (Upperdniestrian/Galician dialect group, Тисів village, Ivano-Frankivsk province, Ukraine): compiled by S. Nikolaev, revised by A. Dybo & A. Kassian. Sources:
    - Field records by S. Nikolaev, 1990s.
- Ukrainian Bogdan (Hutsul dialect group, Богдан village, Zakarpattia province, Ukraine): compiled by S. Nikolaev, revised by A. Dybo & A. Kassian. Sources:
    - Field records by S. Nikolaev, 1990s.
- Belarusian Literary: compiled by V. Chernov, revised by A. Dybo. Sources:
    - Kolas, Ya. et al. (ed.). 2002. *Russko-belorusskij slovar'*. Minsk: Belaruskaya entsyklapedyya. Vol. 1–3.
    - Krapiva, K. (ed.). 2003. *Belaruska-ruski slounik*. 3rd ed. Minsk: Belaruskaya Entsyklapedyya. Vol. 1–3.
    - Tsykhun, G. A. (ed.). 1978. *Etymalagichny sloynik belaruskaj movy.* Minsk: Belaruskaya navuka. Vol. 1–11.

South Slavic

- Bulgarian Literary: compiled by V. Chernov, revised by A. Dybo & A. Kassian. Sources:
    - Bernstein, S. B. 1994. *Bolgarsko-russkij slovar'*. Moscow: Gos. izd-vo inostrannykh i natsional'nykh slovarej.
    - Chukalov, S. 1981. *Russko-bolgarskij slovar'*. Moscow: Russkij yazyk.
    - Georgiev, Vl. (ed.). 1971–2002. *Balgarski etimologichen rechnik*. Sofia: Prof. Marin Drinov. Vol. 1–6.

- Macedonian Literary: compiled by V. Chernov, revised by A. Dybo & A. Kassian. Sources:
  - Field records of Skopje koine by A. Evdokimova in Skopje, 2000s.
  - Koneski, B. (ed.). 1961–1966. *Rečnik na makedonskiot jazik: so srpskohrvatski tolkuvanja*. Skopje: Institut za makedonski jazik. Vol. 1–3.
  - Tolovski, D., Illich-Svitych, V. 1963. *Makedonsko-ruski rechnik*. Moscow: Gos. izd-vo inostrannykh i natsional'nykh slovarej.
- Macedonian Western (based on two dialects, which belong to the Western dialect group: Radozhda-Vevchani and Dihovo): compiled by A. Kassian. Sources:
  - Groen, B. M. 1977. *A structural description of the Macedonian dialect of Dihovo: phonology, morphology, texts, lexicon*. Lisse: Peter de Ridder Press.
  - Hendriks, P. 1976. *The Radožda-Vevčani dialect of Macedonian: structure, texts, lexicon*. Lisse: Peter de Ridder Press.
- Serbo-Croatian Literary: compiled by V. Chernov, revised by A. Dybo & A. Kassian (Zagreb phonetic variants are separated by comma). Sources:
  - Field records by A. Evdokimova in Zagreb, 2000s.
  - Ivanović, S., Petranović, I. 1981. *Russko-serbskokhorvatskij slovar'*. Moscow: Russkij yazyk.
  - *Rečnik srpskohrvatskoga knjizevnog jezika*. Novi Sad / Zagreb, 1967–1976. Vol. 1–6.
  - Skok, P. 1971–1973. *Etimologijski rječnik hrvatskoga ili srpskoga jezika*. Zagreb: Jugoslavenska akademija znanosti i umjetnosti. Vol. 1–3.

West Slavic

- Polish Literary: compiled by V. Chernov, revised by A. Dybo & A. Kassian. Sources:
  - Brückner, A. 1985. *Słownik etymologiczny języka polskiego*. Warsaw: Wiedza Powszechna.
  - Hessen, D., Stypuła, R. 2001. *Wielki słownik polsko-rosyjski*. Warszawa: Wiedza Powszechna. Vol. 1–2.
  - Karłowicz, J. et al. (ed.). 1900–1927. *Słownik języka polskiego*. Warsawa: Nakł. prenumeratorów i Kasy im. Mianowskiego. Vol. 1–8.

- Mirowicz, A. et al. 2001. *Wielki słownik rosyjsko-polski*. Warszawa: Wiedza Powszechna. Vol. 1–2.

- Kashubian Literary: compiled by A. Kassian. Sources:

  - Gołąbek, E. 2012. *Wielki słownik polsko-kaszubski*. Gdańsk: Zrzeszenie Kaszubsko-Pomorskie. Vol. 1–.
  - Sychta, B. 1967–1976. *Słownik gwar kaszubskich*. Wrozław. Vol. 1–7.
  - Trepczyk, J. 1994. *Słownik polsko-kaszubski*. Gdańsk: Zrzeszenie Kaszubsko-Pomorskie. Vol. 1–2.

- Czech Literary: compiled by V. Chernov, revised by A. Kassian. Sources:

  - Kopecký, L. V. et al. (ed.). 1976. *Česko-ruský slovník*. Praha / Moscow: Russkij yazyk. Vol. 1–2.
  - Machek, V. 1968. *Etymologický slovník jazyka českého*. Praha: Československá akademie věd.
  - Rejzek, J. 2001. *Český etymologický slovník*. Praha: Leda.
  - Vlček, J. 1974. *Russko-cheshskij slovar'*. Moscow: Russkij yazyk.

- Slovak Literary: compiled by V. Chernov, revised by A. Dybo & A. Kassian. Sources:

  - Isačenko, A. V. 1950–1957. *Slovensko-ruský prekladový slovník*. Bratislava: Slovenská akadémia vied a umení. Vol. 1–2.
  - Peciar, Š. 1959–1968. *Slovník slovenského jazyka*. Vol. 1–6. Bratislava: Vydavatelstvo slovenskej akadémie vied.

- Upper Sorbian Literary (Upper Lusatian): compiled by V. Chernov, revised by A. Kassian. Sources:

  - Schuster-Šewc, H. 1978–1989. *Historisch-etymologisches Wörterbuch der ober- und niedersorbischen Sprache*. Bautzen: VEB Domowina-Verlag.
  - Trofimovich, K. K. 1974. *Verkhneluzhitsko-russkij slovar'*. Moscow / Bautzen: Russkij yazyk / Domowina.

- Lower Sorbian Literary (Lower Lusatian): compiled by V. Chernov, revised by A. Kassian. Sources:

  - Schuster-Šewc, H. 1978–1989. *Historisch-etymologisches Wörterbuch der ober- und niedersorbischen Sprache*. Bautzen: Domowina.
  - Starosta, M. 1999. *Dolnoserbsko-nemski slownik. Niedersorbisch-deutsches Wörterbuch*. Bautzen: Domowina.

Baltic (East Baltic subgroup)

- Lithuanian Literary: compiled by V. Chernov, revised by A. Dybo & A. Kassian. Sources:
  - Baronas, V., Galinis, V. 1967. *Rusų-lietuvių kalbų žodynas*. Vilnius: Mintis. Vol. 1–2.
  - Fraenkel, E. 1962. *Litauisches etymologisches Wörterbuch*. Heidelberg: C. Winter. Vol. 1–2.
  - Lyberis, A. 2001. *Lietuvių rusų kalbų žodynas*. 3rd ed. Vilnius: Mokslo ir enciklopedijų leidybos institutas.
  - Smoczyński, W. 2007. *Lietuvių kalbos etimologinis žodynas*. Vilnius: Vilniaus universitetas.
- Latvian Literary: Compiled by V. Chernov, revised by A. Dybo & A. Kassian. Sources:
  - Karulis, K. 1992. *Latviešu* etimoloģijas *vārdnīca*. Rīga: Avots. Vol. 1–2.
  - *Latviešu-krievu vārdnīca*. Rīga: Liesma / Avots, 1979–1981. Vol. 1–2.
  - Mozere, R., Millere, A. 2002. *Angļu-latviešu, latviešu-angļu vārdnīca*. Rīga: Zvaigzne ABC.

Outgroup

- German Literary: compiled by V. Chernov, revised by A. Kassian. Sources:
  - Kluge, Fr. 1995. *Etymologisches Wörterbuch der deutschen Sprache*. 24th ed. Berlin: Walter de Gruyter.
  - Leping, A. A. 1954. *Russko-nemetskij slovar'*. Moscow: Gos. izd-vo inostrannykh i natsional'nykh slovarej.

Due to some reasons (e.g., lexicographic incompleteness and dialectal diversity), we prefer not to include extinct languages, such as Old Church Slavonic, Polabian or Old Prussian, in the analysis. Note that exclusion of extinct languages particularly implies that we are only dealing with the East Baltic cluster of the Baltic group. Additionally, Modern Slovenian was intentionally excluded from the dataset, for which see below. For tree rooting, the 110-item wordlist of the German literary language has been introduced as an outgroup.

Geographical distribution of extant Slavic and East Baltic lects used in the study is depicted in Fig. A in S2 File.

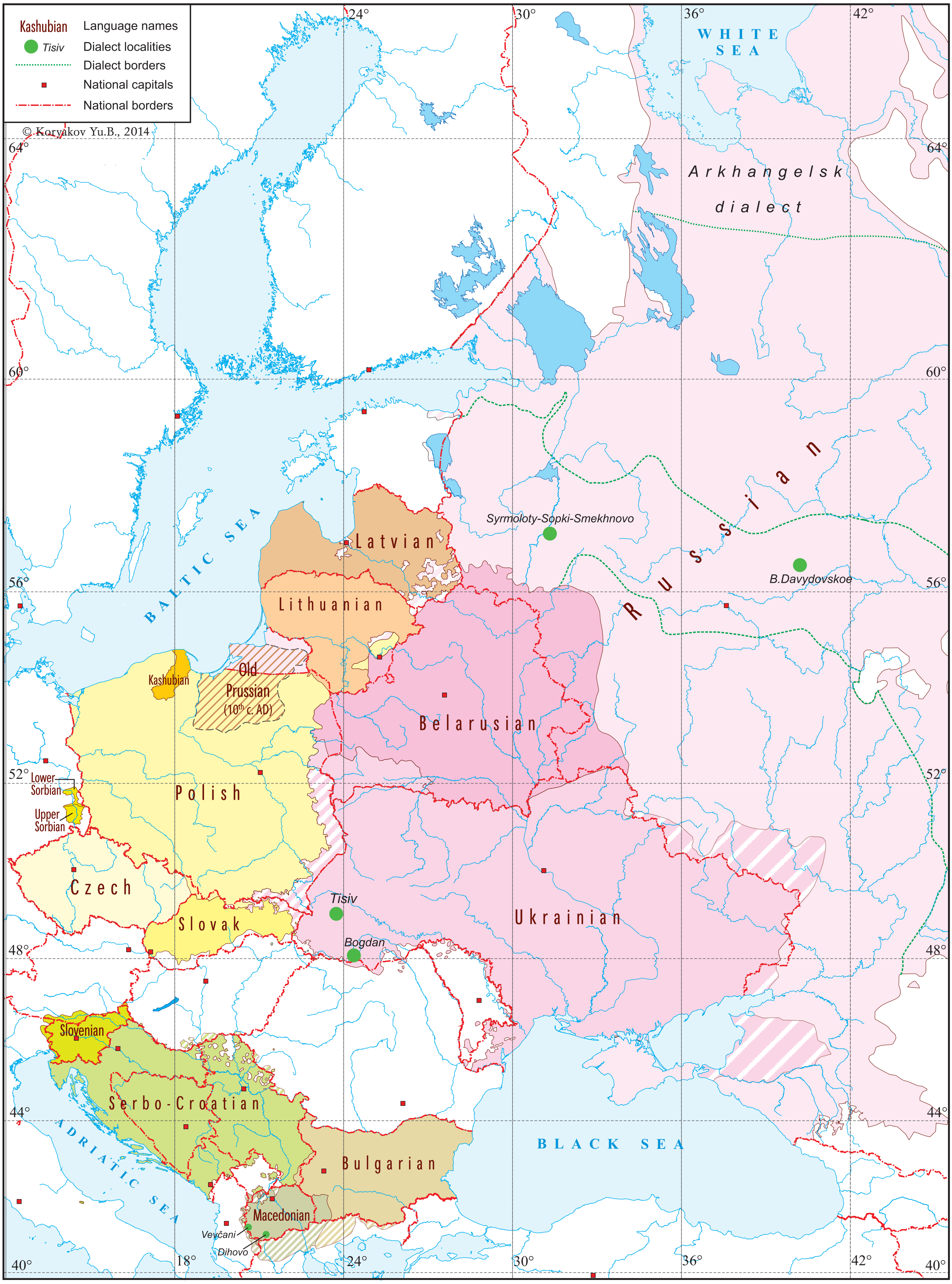

Fig. A in S2 File. Geographical distribution of extant Slavic and East Baltic languages and dialects used in the study. Map was prepared by Yuri Koryakov.

The 110-item set consists of 100 “classical” Swadesh words plus 10 additional words from S. Yakhontov’s 100-wordlist (#101–110 below), taken from the second part of the Swadesh 200-item wordlist, see Burlak & Starostin 2005: 12–13; G. Starostin 2010 for details. Lexical slots are filled in accordance with the semantic specification of the Swadesh items as proposed in Kassian et al. 2010 and currently used in the *Global Lexicostatistical Database* (GLD) project (G. Starostin 2011–2015). The GLD 110-item wordlist runs as follows:

1.	all	23.	eat	45.	know	67.	road	89.	tooth
2.	ashes	24.	egg	46.	leaf	68.	root	90.	tree
3.	bark	25.	eye	47.	lie	69.	round	91.	two
4.	belly	26.	fat n.	48.	liver	70.	sand	92.	go
5.	big	27.	feather	49.	long	71.	say	93.	warm
6.	bird	28.	fire	50.	louse	72.	see	94.	water
7.	bite	29.	fish	51.	man	73.	seed	95.	we
8.	black	30.	fly v.	52.	many	74.	sit	96.	what
9.	blood	31.	foot	53.	meat	75.	skin	97.	white
10.	bone	32.	full	54.	moon	76.	sleep	98.	who
11.	breast	33.	give	55.	mountain	77.	small	99.	woman
12.	burn tr.	34.	good	56.	mouth	78.	smoke	100.	yellow
13.	nail	35.	green	57.	name	79.	stand	101.	far
14.	cloud	36.	hair	58.	neck	80.	star	102.	heavy
15.	cold	37.	hand	59.	new	81.	stone	103.	near
16.	come	38.	head	60.	night	82.	sun	104.	salt
17.	die	39.	hear	61.	nose	83.	swim	105.	short
18.	dog	40.	heart	62.	not	84.	tail	106.	snake
19.	drink	41.	horn	63.	one	85.	that	107.	thin
20.	dry	42.	I	64.	person	86.	this	108.	wind
21.	ear	43.	kill	65.	rain	87.	thou	109.	worm
22.	earth	44.	knee	66.	red	88.	tongue	110.	year

The Balto-Slavic wordlists, used in the present paper, are not included yet in the GLD database (the work is in progress), but we do not expect that further elaboration would alter the principal topology of the obtained trees.

The 20 wordlists of Balto-Slavic lects along with the Modern German were used to create a lexicostatistical matrix (SI Appendix 3), filled with cognation indices (for matrix compilation, see, e.g., Atkinson & Gray 2006: 93–94). Cognation indexes were marked with help of traditional comparative method. We use the standard Balto-Slavic comparative grammar as generally accepted by Indo-Europeanists; see, e.g.:

- Derksen, R. 2007. *Etymological Dictionary of the Slavic Inherited Lexicon*. Leiden / Boston: Brill.
- Stang, Chr. S. 1966. *Vergleichende Grammatik der baltischen Sprachen*. Oslo: Universitetsforlaget.
- Trubachev, O. N. et al. 1974–. *Etimologicheskij slovar' slavyanskikh yazykov*. Moscow: Nauka. Vol. 1–32–.
- Vaillant, A. 1950–1977. *Grammaire comparée des langues slaves*. Lyon / Paris: IAC / Klincksieck. Vol. 1–5.

Loanwords in individual lists are excluded from the analysis (this is a difference from the matrix compilation procedure described in Atkinson & Gray 2006). Both loanwords and undocumented terms, i.e., *lacunae*, are marked as "-1" in STARLING format and "?" in NEXUS format.

S2: 2. Methods

Lexicostatistical trees were produced by several phylogenetic methods.

1. Modified neighbor joining method, designed by S. Starostin for lexicostatistical analysis and implemented in the Starling software (method Starling neighbor joining, hence StarlingNJ); see Burlak & Starostin 2005: 163 ff.; Kassian 2015 for details. In the present paper, the threshold, below which the averaging starts, is not 70%, but 75% that is the default value in the last versions of Starling. The StarlingNJ tree was produced in the Starling software v.2.5.3 (see S. Starostin 1993/2007; Burlak & Starostin 2005: 270 ff.) from the lexicostatistical database, which represents a multistate matrix with synonymy allowed (the multistate matrix is available as SI Appendix 3). The allowed synonymy means that when the same Swadesh slot is occupied by more than one word, i.e., by several synonyms, all possible pairs

of involved words between two languages are compared within this slot: if there is at least one matching pair, the whole slot is treated as a match. The non-parametric bootstrap test was performed (10 000 pseudoreplicates). The hierarchical agglomerative clustering produces, by its very definition, a rooted tree. For node dating, the so-called "experimental method" was applied, according to which each Swadesh item possesses an individual relative index of stability (S. Starostin 2007a; G. Starostin 2010). Dates of the nodes were established by strict molecular clocks, see S. Starostin 1989/2007; S. Starostin 1999/2000; Novotná and Blažek 2007; Balanovsky et al. 2011 on scale calibration and further details. For linguistic time scale, we accept that 0 YPB = AD 2000; since the present study is first of all focused on the Slavic phylogeny and temporal reconstruction, we restrict our date scale to the relevant time depth, 2500 YBP. The tree was visualized in Starling and then manually redrawn for best appearance.

2. Standard neighbor joining method (hence NJ), see Saitou & Nei 1987; Makarenkov et al. 2006: 65–66. The tree was produced in the SplitsTree4 software v.4.13.1 (Huson & Bryant 2006) from the binary lexicostatistical matrix (NEXUS format), which was generated from the original multistate matrix by coding the presence ("1") or absence ("0") of each proto-root (total 364 characters, i.e. proto-roots) in each of the 21 languages; Swadesh items superseded by loanwords or simply not documented are marked as "?" (the binary matrix is available as SI Appendix 3). The non-parametric bootstrap test was performed (10 000 pseudoreplicates). The tree was rooted by the outgroup (the Modern German wordlist). The tree is not dated. The tree was visualized in the FigTree software (v.1.4.1). An additional tree was constructed by the BioNJ method (Gascuel 1997), and appeared to be topologically identical to the NJ one.

3. Unweighted pair group method with arithmetic mean method (hence UPGMA), see Sneath & Sokal 1973: 230–234; Makarenkov et al. 2006: 65–66. The tree was produced in the SplitsTree4 software v.4.13.1 from the binary matrix described above. The non-parametric bootstrap test was performed (10 000 pseudoreplicates). The tree was rooted by the outgroup (the Modern German wordlist). The tree is not dated. The tree was visualized in the FigTree software (v.1.4.1).

4. Markov chain Monte Carlo method under Bayesian framework (hence Bayesian MCMC), see Makarenkov et al. 2006: 68–69, as it was for the first time applied to linguistic data in Gray & Atkinson 2003. The tree was produced in the MrBayes software v.3.2.1 (Huelsenbeck

& Ronquist 2001) from the binary matrix described above. The F81 model was used with rates = gamma. The program was run 4 times using 4 concurrent Markov chains; the Modern German language was marked as an outgroup. Each run produced 5 000 000 tree generations with samples taken every 500 generations. For each run, first 25% tree generations were discarded as a burn-in. The consensus tree was rooted by the outgroup (the Modern German wordlist). The tree is not dated. The tree was visualized in the FigTree software (v.1.4.1).

5. Unweighted maximum parsimony method (hence UMP), see Makarenkov et al. 2006: 66–67. The tree was produced in the TNT software (Willi Hennig Society edition of TNT, v.1.1, 08 May 2013, see Goloboff et al. 2008) from the binary matrix described above by the branch-and-bound ("Implicit enumeration") algorithm. Obligatory binarization of nodes was prohibited ("Collapse trees after the search"); the Modern German language was marked as an outgroup. 1 optimal tree was obtained, for which the non-parametric bootstrap test was performed (1000 pseudoreplicates). The tree was rooted by the outgroup (the Modern German wordlist). The tree is not dated. The tree was visualized in the FigTree software (v.1.4.1).

S2: 3. Results

3.1. Lexicostatistical reconstruction of the Balto-Slavic languages

The following trees were obtained:

Fig B in S2 File. StarlingNJ method with binary nodes only.

Fig. C in S2 File. NJ method.

Fig. D in S2 File. UPGMA method.

Fig. E in S2 File. Bayesian MCMC method.

Fig. F in S2 File. UMP method.

Fig. G in S2 File. Manually constructed consensus tree.

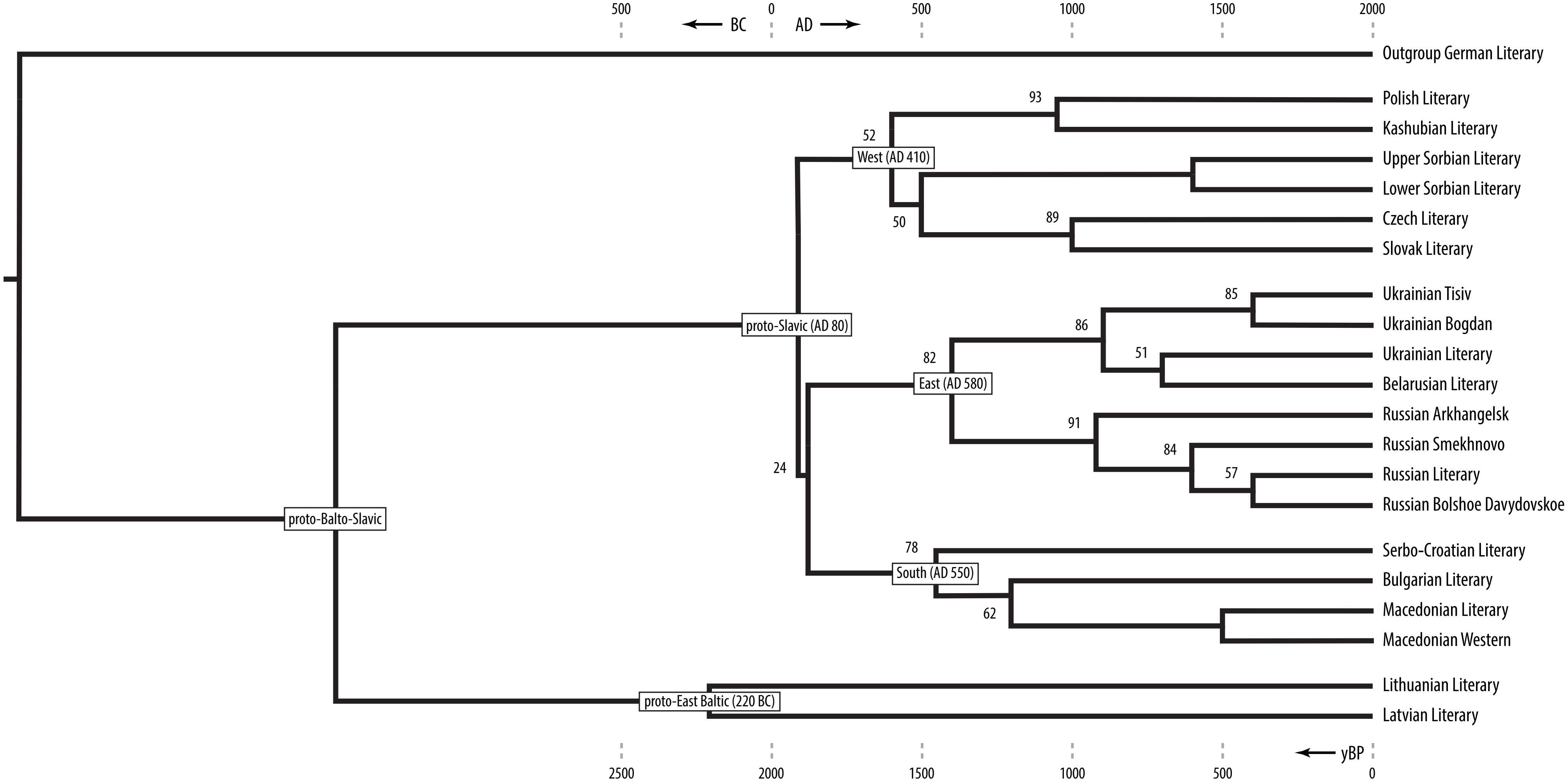

Fig B in S2 File. Dated phylogenetic tree of the Balto-Slavic lects produced by the StarlingNJ method from the multistate matrix (binary nodes only). Bootstrap values are shown near the nodes (not shown for stable nodes with bootstrap value ≥ 95%).

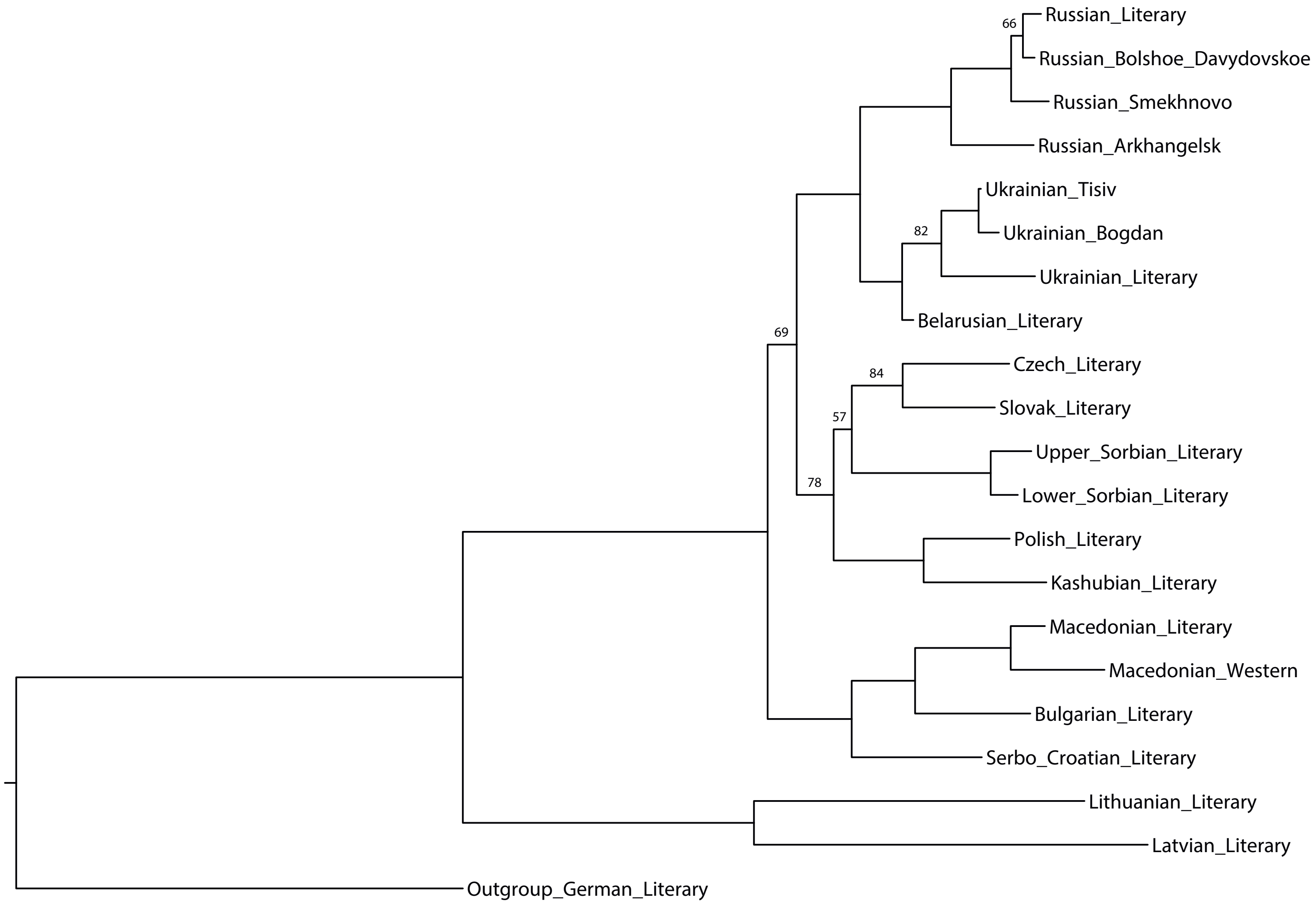

Fig. C in S2 File. Phylogenetic tree of the Balto-Slavic lects produced by the NJ method from the binary matrix in the SplitsTree4 software. Bootstrap values are shown near the nodes (not shown for stable nodes with bootstrap value ≥ 95%). Branch length reflects the relative rate of cognate replacement as suggested by SplitsTree4. The BioNJ method yields the same topology.

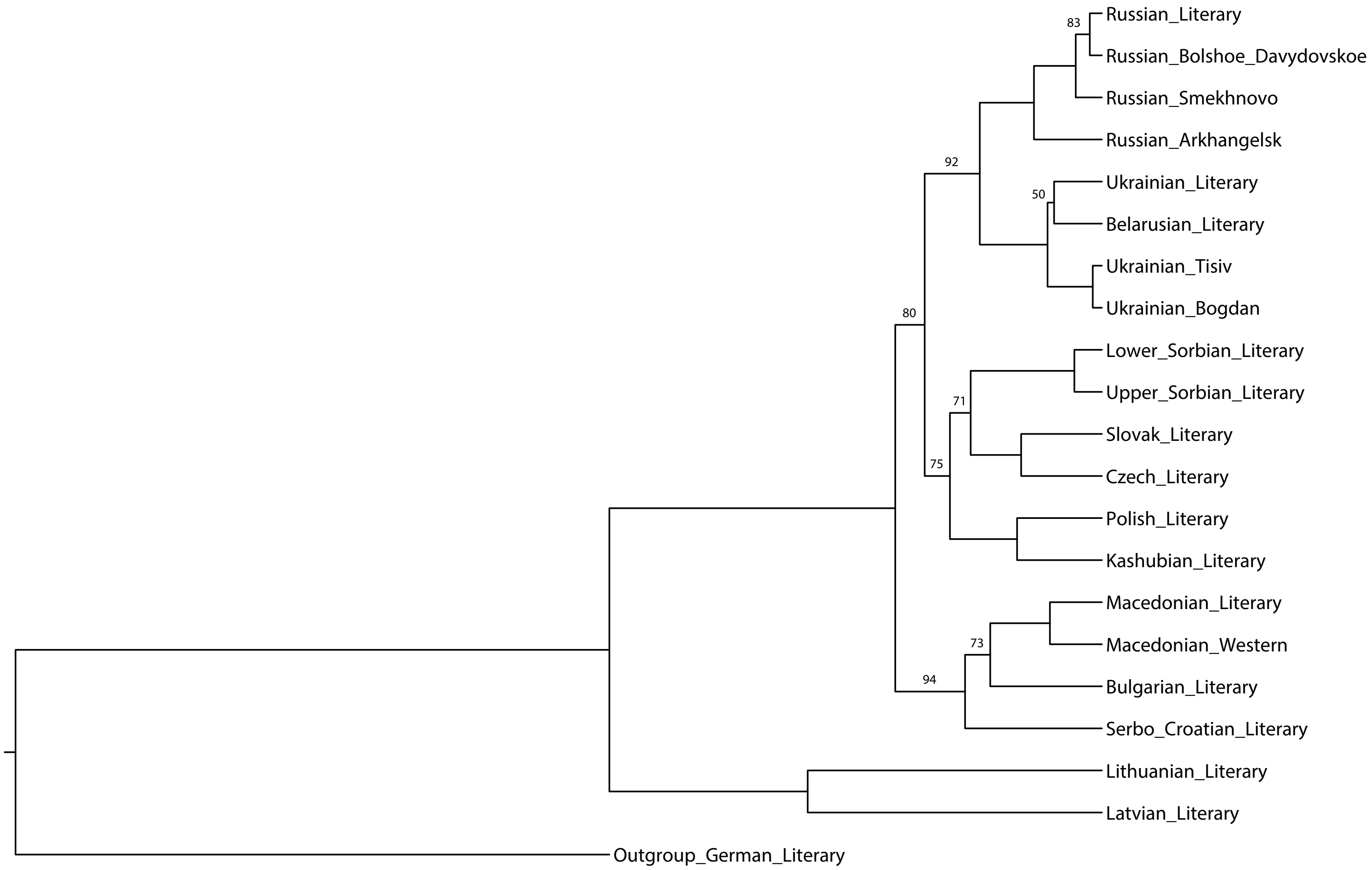

Fig. D in S2 File. Phylogenetic tree of the Balto-Slavic lects produced by the UPGMA method from the binary matrix in the SplitsTree4 software. Bootstrap values are shown near the nodes (not shown for stable nodes with bootstrap value ≥ 95%). Branch length reflects the relative rate of cognate replacement as suggested by SplitsTree4.

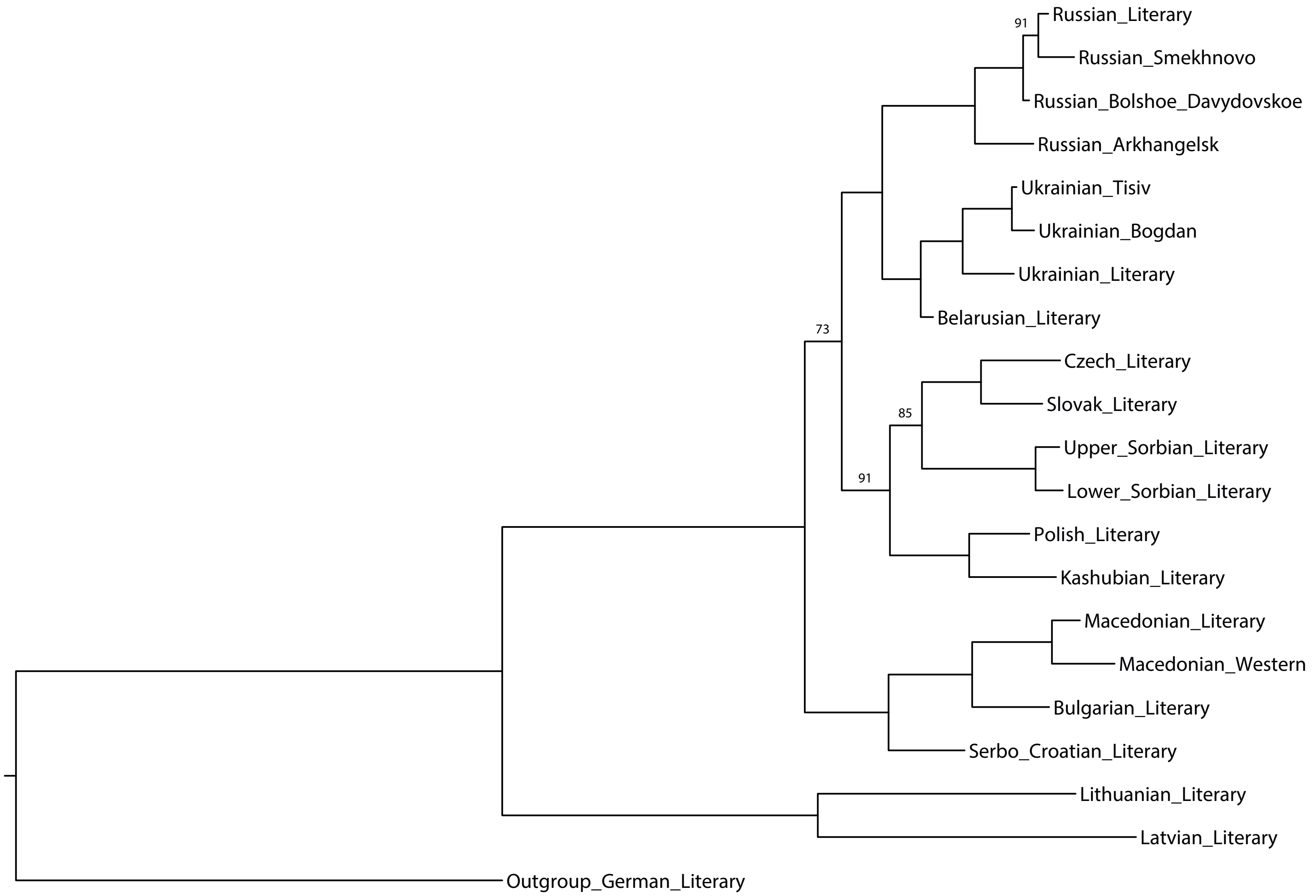

Fig. E in S2 File. Consensus phylogenetic tree of the Balto-Slavic lects produced by the Bayesian MCMC method from the binary matrix in the MrBayes software. Bayesian posterior probabilities are shown near the nodes (not shown for stable nodes with P ≥ 0.95). Branch length reflects the relative rate of cognate replacement as suggested by MrBayes.

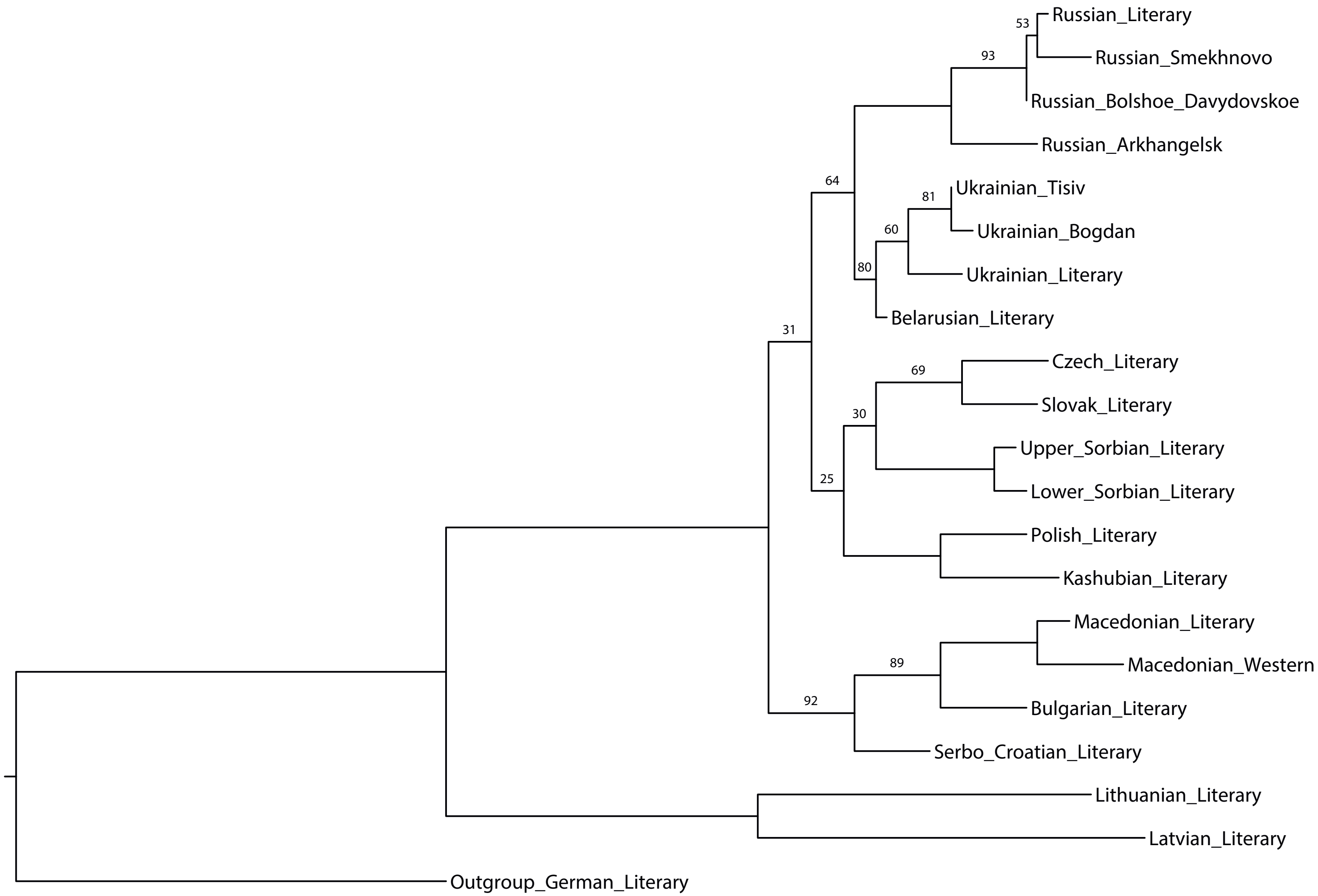

Fig. F in S2 File. Optimal phylogenetic tree of the Balto-Slavic lects produced by the UMP method from the binary matrix in the TNT software. Bootstrap values are shown near the nodes (not shown for stable nodes with bootstrap value ≥ 95%). Branch length reflects the relative rate of cognate replacement as suggested by TNT.

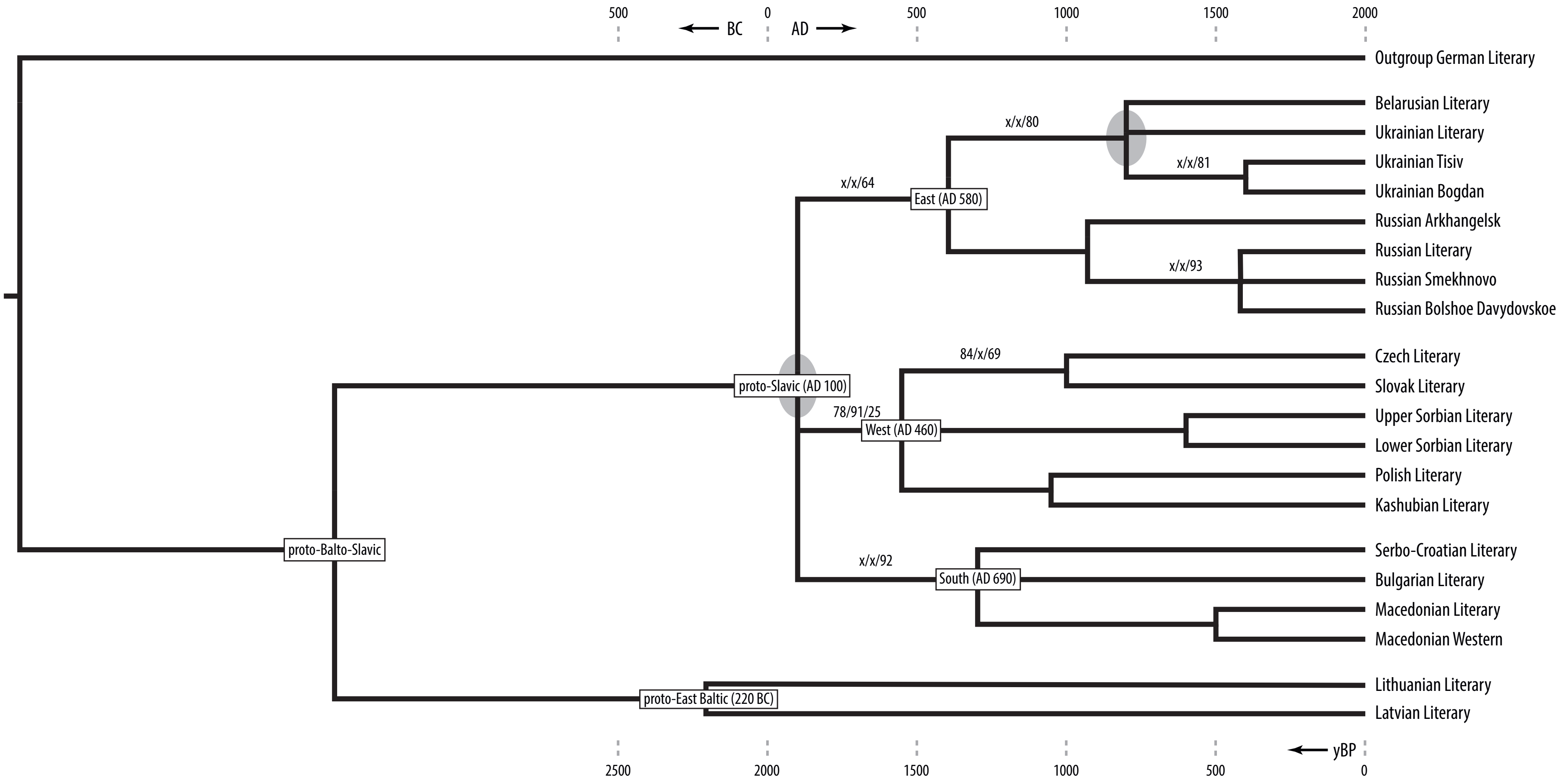

Fig. G in S2 File. Manually constructed consensus phylogenetic tree of the Balto-Slavic lects based on the StarlingNJ, NJ, BioNJ, UPGMA, Bayesian MCMC, UMP methods. Ternary nodes result from neighboring binary nodes, joined together, if the temporal distance between them ≤ 300 years. The gray ellipses additionally mark two joined nodes, which cover binary branchings that differ depending on the method. Probability values are shown in the following sequence: NJ/Bayesian MCMC/UMP (“x” means that P ≥ 0.95 in an individual method; not shown for nodes with P ≥ 0.95 in all methods). StarlingNJ dates are proposed.

All the methods confidently detect the four main clades: East Baltic, East Slavic, West Slavic and South Slavic, and suggest the same principal topology of the taxa within these clades.

Two topological discrepancies between the obtained trees can be noted:

1) StarlingNJ (Fig B in S2 File) differs from all other methods (Fig. C–F in S2 File) in the Proto-Slavic node. StarlingNJ suggests that West Slavic separates first, other methods claim that the first separating clade is South Slavic. According to StarlingNJ (Fig B in S2 File), however, the temporal span between the Proto-Slavic node and the East+South node is nominal and insignificant from the historical point of view (40 years). In turn, other methods (Fig. C–F in S2 File) reveal that probabilities obtained for the distinct East+West clade are not very high (UMP 0.31, Bayesian MCMC 0.73, NJ 0.69, UPGMA 0.8).

2) According to StarlingNJ (Fig B in S2 File) and UPGMA (Fig. D in S2 File), Ukrainian Literary & Belarusian Literary form a distinct clade. On the contrary, NJ (Fig. C in S2 File), Bayesian MCMC (Fig. E in S2 File), UMP (Fig. F in S2 File) suggest that Belarusian Literary separates prior to all Ukrainian lects. Apparently, such a discrepancy is an effect of the mixed dialectal origin and somewhat artificial nature of the Belarusian literary language. The distinct node "Ukrainian Literary + Belarusian Literary" is, however, rather week: StarlingNJ indicates that the time span between the "Proto-Ukrainian-Belarusian" node and the specific "Ukrainian Literary + Belarusian Literary" node is very small from the historical point of view: 180 years; UPGMA suggests that the probability of the node "Ukrainian Literary + Belarusian Literary" is just 0.496. Some other methods, which join all Ukrainian lects in one distinct clade, demonstrate that the probability of such a clade is not very high: UMP 0.6, NJ 0.82.

The resulting consensus tree was obtained by joining discussed above "problematic" binary nodes into two ternary nodes: Proto-Slavic and Ukrainian-Belarusian. It seems, however, reasonable to go further and join any neighboring nodes together if the temporal distance between them is ≤ 300 years as calculated by the StarlingNJ method (Fig B in S2 File). Such a procedure yields three additional ternary nodes (Russian lects except Arkhangelsk; Proto-South Slavic; Proto-West Slavic). Note that the individual binary nodes, covered by these five ternary nodes, normally demonstrate probability < 0.95, as proposed by the methods used. See Fig. G in S2 File for the manually constructed consensus tree with the StarlingNJ dates.

Our consensus tree (Fig. G in S2 File) suggests the following topological and temporal reconstruction of the Balto-Slavic languages. Initial disintegration of proto-Balto-Slavic into proto-Baltic and proto-Slavic took place during the 2nd millennium BC. Proto-Slavic splits into three major clades, East, West, South Slavic around AD100 (1900 YBP). Further diversification of each clade into minor clades (i.e. proto-East Slavic: Ukrainian/Belarusian, Russian; proto-West Slavic: Czech/Slovak, proto-Sorbian, Polish/Kashubian; proto-South Slavic: Serbo-Croatian, Bulgarian, Macedonian) took place during the 5th–7th centuries AD (about 1500–1300 YPB), followed by final shaping of individual languages (1000–500 YBP).

Taken together, our consensus tree (Fig. G in S2 File) agrees with the traditional Slavistic and Indo-Europeanistic views on the structure and history of this language group (see, e.g., Sussex, Cubberley, 2006: 42 f.; Blažek, 2007). In particular, the traditional view is that “the real break-up of Proto-Slavic unity began about the fifth century AD” (Sussex, Cubberley, 2006: 20), despite the accepted fact that in previous centuries, the Proto-Slavs occupied a relatively large area in East Europe (Sussex, Cubberley, 2006: 19) that should imply existence of a dialectal diversity of some kind. Our estimation of AD 100 does not contradict it, since lexicostatistical divergence is the first discrepancy between Swadesh wordlists of two lects, but in normal case, these lects remain fully mutually intelligible and are readily able to share various common innovations. The splits within the East, West and South Slavic clades during the 5th–7th centuries AD fit modern views on historical records and archaeological data which indicate the quick expansion of the Slavs across Europe, the so-called Slavicization of Europe, during the second half of the 1st millennium AD (Sedov 1979; Barford 2001; Curta 2001; Heather 2010).

3.2. The case of the Slovenian language

Modern Slovenian belongs to the South Slavic clade according to the traditional classification of Slavic languages (Sussex, Cubberley, 2006). However, significant linguistic similarities between Slovenian and West Slavic lects have been observed earlier in number of studies. See, for example, on specific ties between Slovenian and West Slavic (e.g., Slovak) or even on support of the mixed South/West origin of Slovenian, e.g., Bezlaj, 2003, Sobolev, 2000, Bernstein, 1961, Stieber, 1972, Lekov, 1958.

Likewise, the Slovenian (Ljubljana koine and literary Slovenian) wordlist, available in our study (see sources below), possesses a substantial number of both South Slavic and West Slavic lexical matches (cf. similar observations in Novotná and Blažek, 2007: 195). Such a mix introduces enough incompatible characters into the input matrix to make the calculation of robust trees impossible. Due to this reason we have deliberately excluded Modern Slovenian from the current analysis. We suggest that one of the possible scenarios is that Slovenian is historically a West Slavic language being influenced by neighboring Serbo-Croatian during the last millennium.

To demonstrate specific ties between Slovenian and South Slavic, on the one hand, and West Slavic, on the other, we calculated a set of NeighborNet phylogenetic networks (Bryant, Moulton, 2004; Makarenkov et al. 2006: 89–90) of Balto-Slavic languages with the use of SplitsTree4 software from the binary matrix described above; the non-parametric bootstrap test was performed with 10 000 pseudoreplicates in each case. Two additional taxa were introduced into the original dataset: Slovenian and Modern Demotic Greek, the latter as an outgroup for the Germanic-Balto-Slavic clade.

- Slovenian Ljubljana (Ljubljana koine): compiled by A. Kassian. Sources:
  - Field records by K. Ogrinc, summer 2014.
- Modern Demotic Greek (Athens koine). Sources:
  - Evdokimova, A., Kassian, A. 2014. *Annotated Swadesh wordlists for Modern Demotic Greek*, based on field records of 2006. In: G. Starostin (ed.). *The Global Lexicostatistical Database*. Moscow/Santa Fe: Center for Comparative Studies at the Russian State University for the Humanities; Santa Fe Institute. Available: http://starling.rinet.ru/new100

The following networks are presented here:

Fig. H in S2 File. Balto-Slavic (without Slovenian) + German.

Fig. I in S2 File. Balto-Slavic (without Slovenian) + Demotic Greek.

Fig. J in S2 File. Balto-Slavic (without Slovenian) + German + Demotic Greek.

Fig. K in S2 File. Balto-Slavic (with Slovenian) + German.

Fig. L in S2 File. Balto-Slavic (with Slovenian) + Demotic Greek.

Fig. M in S2 File. Balto-Slavic (with Slovenian) + German + Demotic Greek.

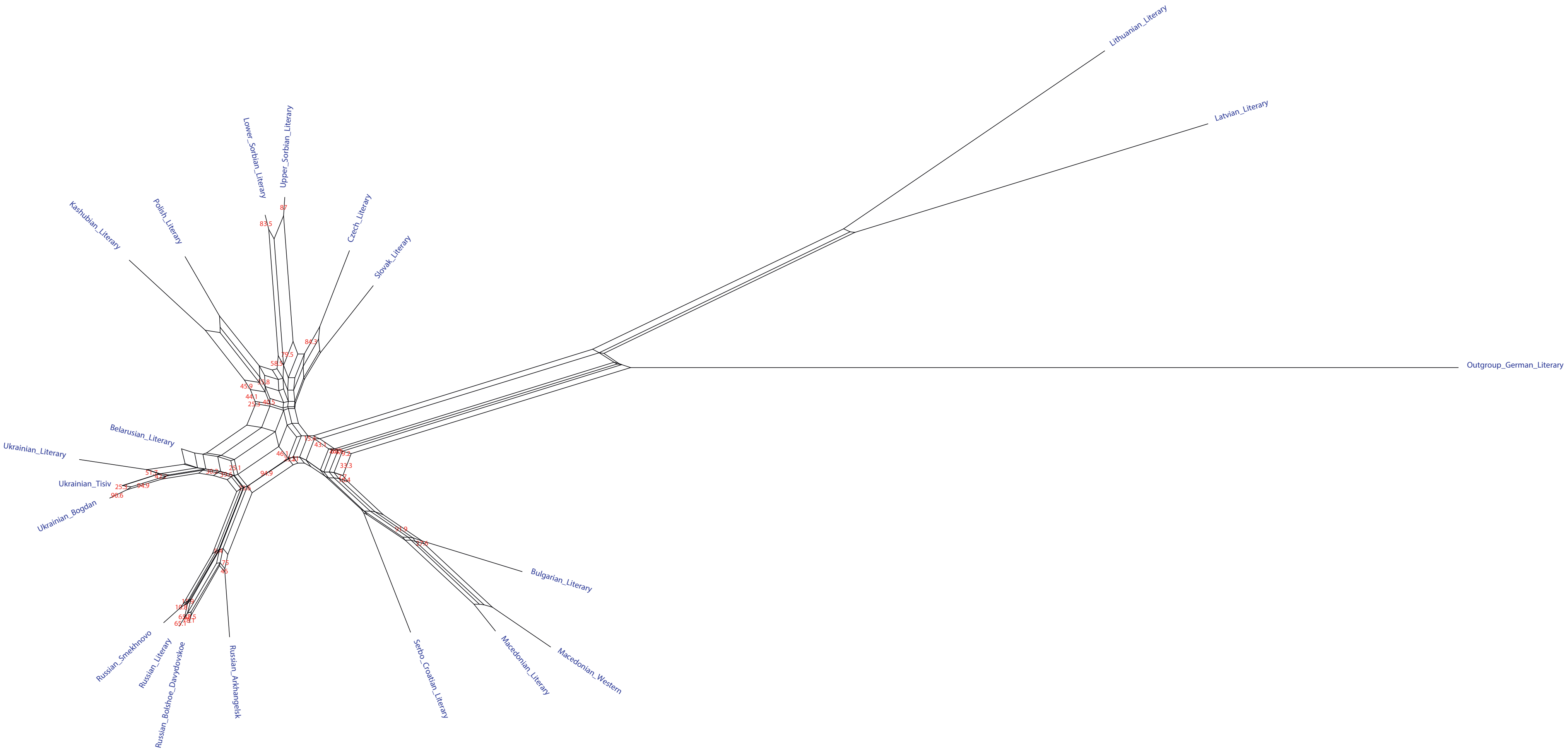

Fig. H in S2 File. NeighborNet network of the Balto-Slavic lects (without Slovenian) + German. Produced in the SplitsTree4 software; bootstrap values are shown near the nodes (not shown for stable nodes with bootstrap value ≥ 95%).

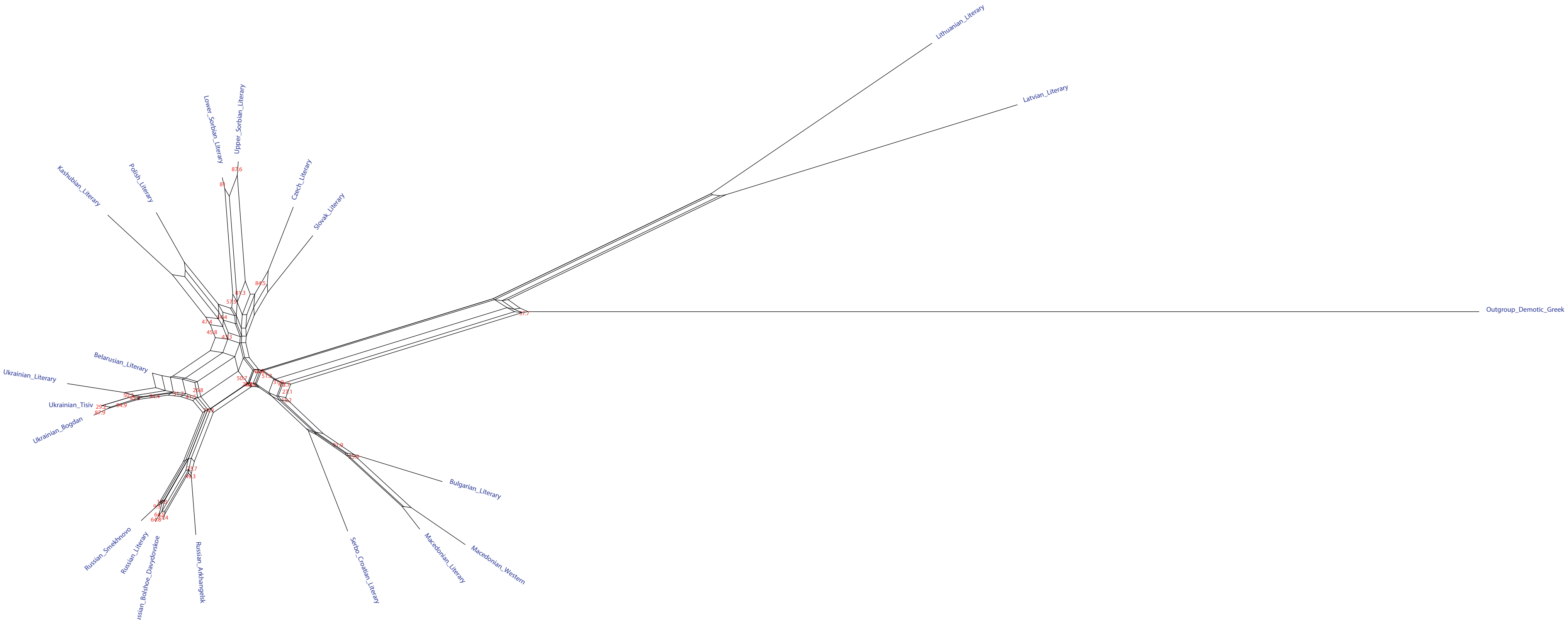

Fig. I in S2 File. NeighborNet network of the Balto-Slavic lects (without Slovenian) + Demotic Greek. Produced in the SplitsTree4 software; bootstrap values are shown near the nodes (not shown for stable nodes with bootstrap value ≥ 95%).

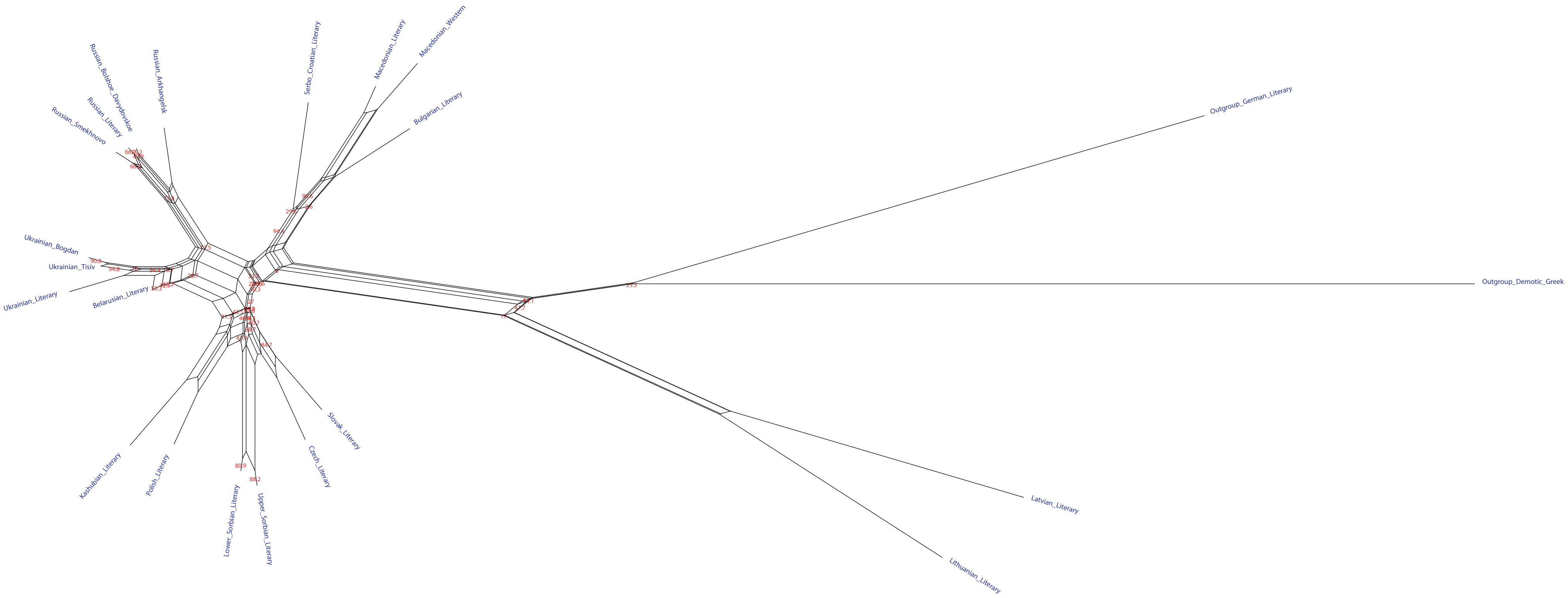

Fig. J in S2 File. NeighborNet network of the Balto-Slavic lects (without Slovenian) + German + Demotic Greek. Produced in the SplitsTree4 software; bootstrap values are shown near the nodes (not shown for stable nodes with bootstrap value ≥ 95%).

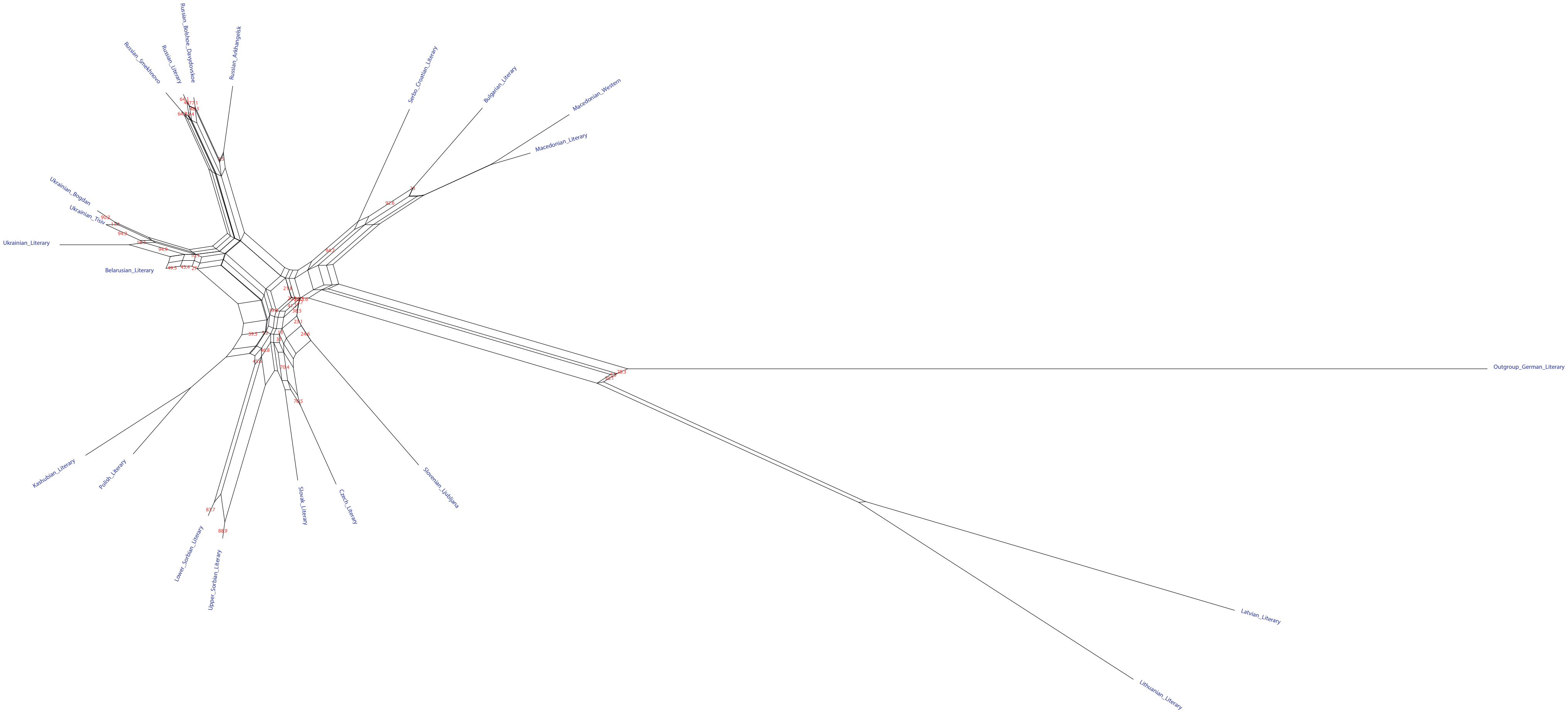

Fig. K in S2 File. NeighborNet network of the Balto-Slavic lects (with Slovenian) + German. Produced in the SplitsTree4 software; bootstrap values are shown near the nodes (not shown for stable nodes with bootstrap value ≥ 95%).

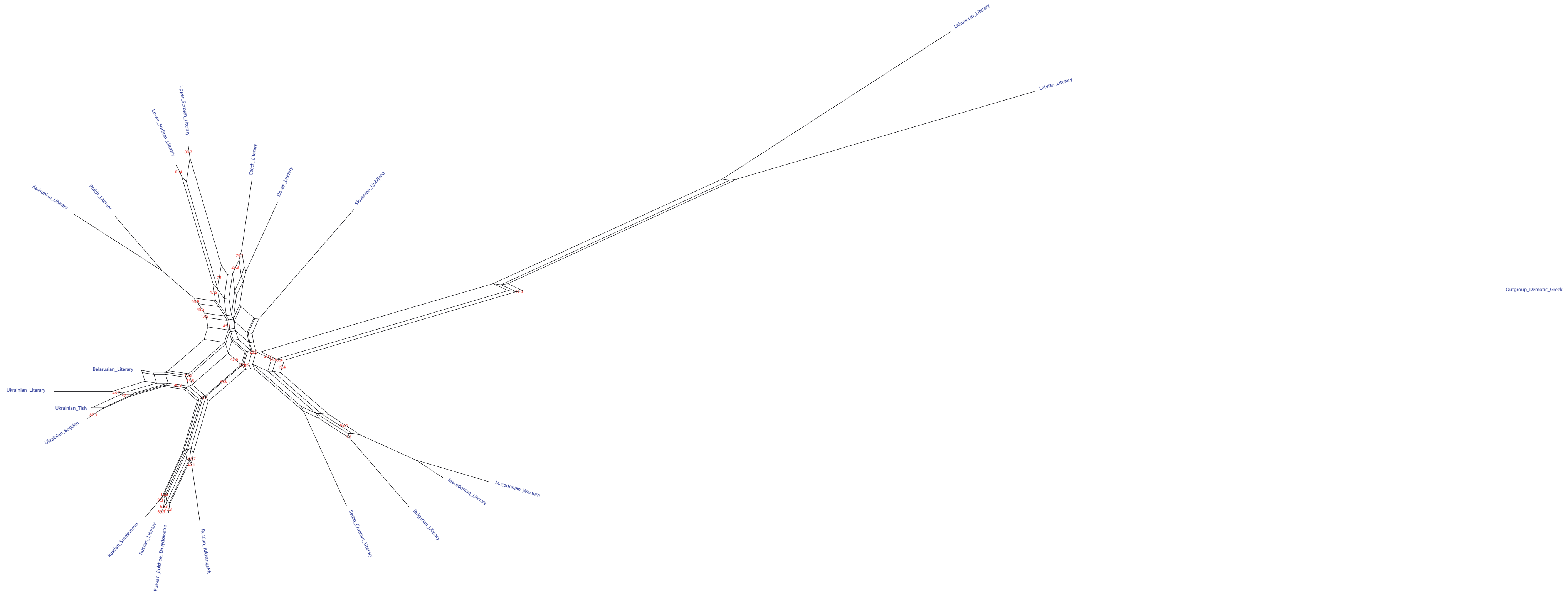

Fig. L in S2 File. NeighborNet network of the Balto-Slavic lects (with Slovenian) + Demotic Greek. Produced in the SplitsTree4 software; bootstrap values are shown near the nodes (not shown for stable nodes with bootstrap value ≥ 95%).

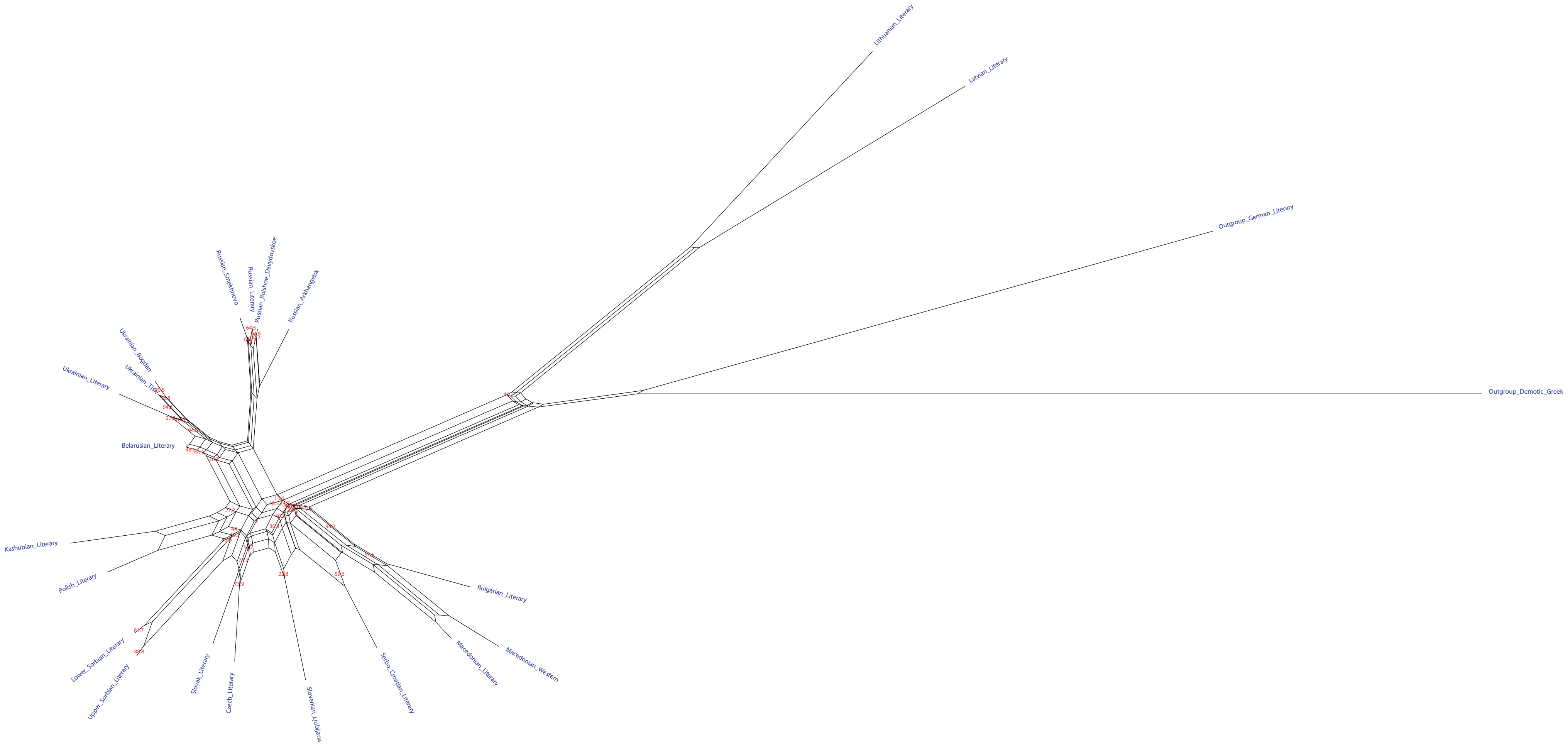

Fig. M in S2 File. NeighborNet network of the Balto-Slavic lects (with Slovenian) + German + Demotic Greek. Produced in the SplitsTree4 software; bootstrap values are shown near the nodes (not shown for stable nodes with bootstrap value ≥ 95%).

Three networks without Slovenian (Fig. H–J in S2 File) reveal the same major clades of Balto-Slavic languages as phylogenetic trees do (Fig. B–G in S2 File) irrespective the out-group used. Incorporation of Slovenian into network analysis reveals following: Slovenian appears to be an independent branch of Slavic languages which is nearly equally close to West and South Slavic, but distant from East Slavic (Fig. K–M in S2 File), thus supporting the putative mixed nature of Modern Slovenian.

Further lexicostatistical investigation of Slovenian dialects, such as in progress in the GLD project, are needed to elucidate the place of Slovenian among Slavic languages.

3.3. Previous formal phylogenetic studies on Slavic languages

Among other formal phylogenetic studies on the Slavic languages, one should mention Novotná and Blažek, 2007 (with an additional overview of the previous Slavic lexicostatistical classifications by M. Čejka, G. J. Vollmer, S. A. Starostin); Gray and Atkinson, 2003; Bouckaert et al., 2012/2013; Müller et al. 2013. The studies make use of different datasets and methodology and suggest somewhat different topological and/or temporal reconstructions of Slavic languages.

Novotná and Blažek, 2007 operate with 100-item Swadesh wordlists analyzed by the StarlingNJ algorithm in the Starling software. Authors' approach to the wordlist compilation differs significantly from the Moscow school principles and especially from the current *Global Lexicostatistical Database* project standards in terms of strictness and rigor (Kassian et al. 2010). Particularly, authors abundantly include (quasi)synonyms (not full synonyms) in the lists, regardless of the synchronic status of a specific word — be it rare, obsolete, dialectal, stylistically marked, semantically dubious (for ancient languages) and so on. Such an uncontrolled inclusion of quasi-synonyms in the input wordlists causes additional lexicostatistical matches between *taxa* that should make reconstructed nodes younger. According to Novotná and Blažek, 2007, the initial split of Proto-Slavic dates back to AD 520. The resulting Slavic tree, offered in Novotná and Blažek, 2007: 201, does not contain evident flaws (it does not mean that all proposed binary branchings are equally likely), but temporal spans between the nodes are short that they can hardly make sense from the historical point of view. E.g., the distance between the West+South node (AD 750) and the West node (AD 900) is just 150 years.

In two papers by R. Gray and Q. Atkinson's team (Gray and Atkinson, 2003; Bouckaert et al., 2012/2013), Indo-European phylogenetic trees are offered, which are based on 200-item wordlists analyzed by the Bayesian MCMC algorithm. 87 Indo-European wordlists taken from the database Dyen et al., 1997 (plus three extinct languages) are used in Gray and Atkinson, 2003. Further this dataset was extended up to 103 wordlists in Bouckaert et al., 2012/2013.

After careful inspection of the Balto-Slavic wordlists from the Indo-European lexicostatistical database (Dyen et al., 1997, http://www.wordgumbo.com/ie/cmp/, compiled in the 1960s) we found that these are not free from errors. For example, within the 110-item subset that used in our study, there are, in our opinion, at least 10 etymologically important, i.e., relevant for phylogenetic analysis, errors in Russian (two Russian lists are offered in Dyen et al., 1997, which are labeled as "Russian" & "Russian P"):

- 'big': "VELIKIJ"; actually the common modern meaning of *velikij* is 'great'.
- 'cloud': "TUCA"; actually *tuča* has the specific meaning 'black cloud, rain cloud'.
- 'cloud': "OBLAKO"; *oblako* 'cloud' is not an inherited form, but a transparent Church Slavonic loanword.
- 'dog': "SOBAKA"; *sobaka* is not an inherited term, but a loanword of Iranian origin.
- 'good': "DOBRYJ"; actually the common modern meaning of *dobryj* is 'kind'.
- 'man (male human being)': "CELOVEK"; actually the basic meaning of *čelovek* is 'person, human being'.
- 'person, human being': "LICO"; actually a generic neutral term is *čelovek*, whereas *lico* is its more rare synonym, used in official language.
- 'road': "PUT"; actually *put'* means 'path' (normally metaphorically).
- 'seed': "ZERNO"; actually the basic meaning of *zerno* is 'grain'.
- 'year': "LETA"; actually pluralia tantum *leta* means 'age, years'.

To our opinion, at least 24 etymologically important errors in the Belarusian 110-item subset (two Belarusian lists are offered in Dyen et al., 1997, which are labeled as "Byelorussian" & "Byelorussian P"):

- ‘belly’: “BRUXA”; apparently the noun *bruxa* ‘belly’ does not exist in modern language.
- ‘burn [intrans.]’: “PALIC”; actually *palic’* means ‘to burn [trans.]’.
- ‘cloud’: “VOBLAKA”; actually *voblaka* ‘cloud’ is not an inherited form, but a transparent Church Slavonic loanword.
- ‘cloud’: “XMARA”; actually *xmara* has the specific meaning ‘black cloud, rain cloud’.
- ‘cold (of weather)’: “S'CJUDZENA”; *sc’uʒ’ony* indeed means ‘cold (of weather)’, but it is a rare and marginal word.
- ‘dog’: “SABAKA”; actually *sabaka* is not an inherited term, but a loanword of Iranian origin.
- ‘dog’: “POS”; apparently the noun *p’os* ‘dog’ does not exist in modern language.
- ‘earth’: ‘HLEBA’; actually *ɣleba* indeed means ‘earth (soil)’, but it is a technical term similar to English *soil* rather than an everyday word.
- ‘fat’: “TUK”; actually *tuk* is a rare and marginal term for ‘fat, dissolved fat’, probably of Polish origin.
- ‘fat’: “TLUSC”; actually *tlušč* ‘fat’ is not an inherited form, but a transparent Polish loanword.
- ‘foot’: “STAPA”; actually *stapa* ‘foot’ is a very rare literary word.
- ‘hear’: “SLUCHAC”; actually *sluchac’* means ‘to listen’.
- ‘lie’: “ABKLADAC’”; actually *abkladac’* means ‘to put round, to edge’.
- ‘liver’: “VANTROBA”; actually rare and marginal *vantroba* ‘intestines, liver’ is not an inherited form, but a transparent Polish loanword.
- ‘man’: “CALAVEK”; actually the basic meaning of *čalavek* is ‘person, human being’.
- ‘many’: “SMAT”; actually *šmat* ‘many’ is not an inherited form, but a Polish loanword.
- ‘neck’: “KARAK”; actually *karak* means specifically ‘nape of the neck’ and represents a Polish loanword.
- ‘person, human being’: “ASOBA”; actually a generic neutral term is *čalavek*, whereas *asoba* is its more rare synonym, used in official language.
- ‘red’: “CYRVONY”; actually *čyrvony* ‘red’ is not an inherited form, but a transparent Polish loanword.

- ‘road’: “SLJAX”; actually *šl’ax* means ‘path’ or specifically ‘high road’ and represents a Polish loanword.
- ‘see’: “BACYC”; actually *bačyc’* ‘to see’ is not an inherited form, but a Polish loanword.
- ‘skin’: “SKURA”; actually *skura* ‘skin’ is not an inherited form, but a transparent Polish loanword.
- ‘THAT’: “HETY”; actually *ɣety* means ‘this’.
- ‘tree’: “DREVA”; actually *dreva* ‘tree’ cannot be an inherited form, apparently a Polish or Church Slavonic loanword although details are not entirely clear.

To our opinion, at least 9 etymologically important errors in the Polish 110-item subset (two Polish lists are offered in Dyen et al., 1997, which are labeled as “Polish” & “Polish P”):

- ‘bite’: “GRYZC”; actually the main meaning of *gryźć* is ‘to gnaw, nibble’, it is not the basic verb for ‘to bite’.
- ‘cloud’: “CHMURA”; actually *chmura* has the specific meaning ‘black cloud, rain cloud’, rather than neutral generic ‘cloud’.
- ‘cold (of weather)’: “CHLODNY”; actually *chłodny* means specifically ‘cool, chilly’ rather than neutral generic ‘cold’.
- ‘foot’: “STOPA”; actually *stopa* ‘foot’ is a rare word, it is not the basic term for this meaning.
- ‘man’: “CZLOWIEK”; actually the basic meaning of *człowiek* is ‘person, human being’.
- ‘person, human being’: “OSOBA”; actually a generic neutral term is *człowiek*, whereas *osoba* is its more rare synonym, used in official language.
- ‘seed’: “ZIARNO”; actually the basic meaning of *ziarno* is ‘grain’.
- ‘snake’: “ZMIJA”; actually the main meaning of *żmija* is ‘viper’.
- ‘worm’: “CZERW”; actually the main meaning of *czerw* is ‘caterpillar’.

To our opinion, at least 3 etymologically important errors in the Lithuanian 110-item subset (two Lithuanian lists are offered in Dyen et al., 1997, which are labeled as “Lithuanian ST” & “Lithuanian O”):

- ‘person, human being’: “ASMUO”; actually a generic neutral term is *žmogus*, whereas *asmuo* is its more rare synonym, used either officially or ironically.

- ‘skin’: “SKURAS”; actually *skuras* (if exists!) is a transparent Polish loanword.
- ‘to walk, go’: “VAIKSCIOTI”; actually a frequent generic verb of going is *eiti*, whereas *vaikščioti* normally means ‘to take a walk’, and more rarely ‘to walk, go’.

As said above, additional Indo-European lects were added in Bouckaert et al. 2012/2013, and the whole dataset was somewhat revised (it is available as “Indo-European Lexical Cognacy Database” at http://ielex.mpi.nl/, accessed July 2014). Additional lexicographical sources used in Bouckaert et al., 2012/2013 vary in their quality. On the one hand, there are indeed some full-fledged synchronic dictionaries, on the other hand, the authors readily resort to such sources as anonymous Swadesh wordlists from the on-line English version of *Wikipedia: the free encyclopedia* (http://en.wikipedia.org/; for some lects, like, e.g., Friulian, *Wikipedia* appears to be the only lexicographical source) or on-line educational publications (e.g., *Ancient Greek tutorials*: http://socrates.berkeley.edu/~ancgreek/ancient_greek_start.html).
It appears to us, however, that along with improvements of the Indo-European lexicostatistical database Dyen et al., 1997, some additional etymological errors were included. For example, within our 110-item subset, at least 1 etymologically important error has been added into the Polish list:

- ‘moon’: “miesiąc”; this Polish noun only means ‘month’ in modern language, not ‘moon’.

At least 5 etymologically important errors have been added into the Lithuanian 110-item subset (“Lithuanian ST”):

- ‘burn’: “svelù”; actually *svìlti*, pres. *svylù* (dialectal *svelù*) means ‘to be (slightly) burnt (said of food)’.
- ‘say’: “tarti”; actually *tarti* means ‘to pronounce, articulate’.
- ‘skin’: “plėnė”; actually *plėnė* means ‘membrane, peel, pellicle’.
- ‘tree’: “drevė”; actually *drevė* means ‘tree hollow’.
- ‘tree’: “derva”; actually *derva* means ‘pitch, tar, resin’.

Etymological analysis, accepted in Bouckaert et al. 2012/2013 and in the “Indo-European Lexical Cognacy Database”, could also be questioned. It seems that the main tendency is “lumping”, i.e. treating etymologically unrelated forms as cognate. For example, within the 110-item subset, there are, in our opinion, at least 3 errors in etymologization of Belarusian words:

- ‘fat’: “TUK, TLUSC”; Belarusian *tuk* and *tlušč*, listed together in the cognate class #1225 at http://ielex.mpi.nl/cognate/1225/, are etymologically unrelated, containing two distinct Slavic roots (note that apparently both Belarusian forms are Polish loanwords).
- ‘hear’: “SLUCHAC, CUC’”; Belarusian *sluxac’* and *čuc’*, listed together in the cognate class #598 at http://ielex.mpi.nl/cognate/598/, are etymologically unrelated, containing two distinct Slavic roots (note that one of them actually means ‘to listen’, not ‘to hear’).
- ‘say’: “KAZAC”; the Belarusian verb *kazac’* is etymologically unrelated to Polish *rzec* ‘to say’ and other Slavic verbs listed in the cognate class #1169 at http://ielex.mpi.nl/cognate/1169/.

At least 2 errors in etymologization of Polish words (110-item subset):

- ‘come’: “przyjść, przychodzić”; these Polish verbs, listed together in the cognate class #1119 at http://ielex.mpi.nl/cognate/1119/, indeed form a suppletive perfective/imperfective paradigm with the generic meaning ‘to come’, but they are etymologically unrelated, containing two distinct Slavic roots.
- ‘seed’: “ZIARNO”; Polish *ziarno*, Russian *zerno*, etc. (actually meaning ‘grain’) are etymologically unrelated to Belarusian *sem’a* and other Slavic forms for ‘seed’, listed together in the cognate class #289 at http://ielex.mpi.nl/cognate/289/.

But examples for “splitting”, i.e., treating cognate forms as etymologically unrelated, can also be found. Cf. the following instance outside the Balto-Slavic group:

- under the entry ‘blood’, two distinct cognate sets are postulated. One (#859, http://ielex.mpi.nl/cognate/859/) includes Hittite *ešḫar*, Tocharian *ysār, yasar*, and so on; the other (#574, http://ielex.mpi.nl/cognate/574/) includes Old Indic *ásr̥k*, Armenian *ariun*, Latvian *asins* and so on. The authors claim that the two sets “may ultimately be cogn.” to each other, but it is “doubted by Buck”, so they prefer to divide the aforementioned forms into two unrelated groups. The authors refer to p. 206 of the well-known C. D. Buck’s *A dictionary of selected synonyms in the principal Indo-European languages* (Chicago: The University of Chicago, 1949/1988). In fact, however, this reference is misleading, since Buck as well as all other Indo-Europeanists have no doubt that the above forms are etymologically re-

lated (e.g., Buck explicitly lists Hittite *ešḫar*, Tocharian *ysār, yasar*, Old Indic *ásr̥k* and Armenian *ariun* in the same paragraph).

Regrettably, we believe that the total amount of incorrect forms in Dyen et al., 1997 and, correspondingly, in "Indo-European Lexical Cognacy Database" is critical, and any phylogenetic study based on these datasets should be treated with caution.

If we take the Belarusian-Polish pair, the aforementioned lexicographic and etymological inaccuracies and errors in the datasets, used by Gray and Atkinson's team, provide 18 parasitic matches between the Belarusian and Polish 110-item wordlists (see above 'belly', 'burn', 'cloud', 'cloud', 'cold', 'dog', 'fat', 'foot', 'hear', 'liver', 'man', 'person', 'red', 'say', 'skin', 'snake', 'tree', 'worm'). I.e., *ca.* 35 parasitic Belarusian-Polish matches are expected if we extrapolate it to the whole 200-item wordlist. As a result, the Slavic section of the Indo-European tree in Gray and Atkinson, 2003 indeed contains the South Slavic and East Slavic lects as two distinct clades, but it is not the case of West Slavic taxa, since the East Slavic clade turns out to be inserted in the very midst of West Slavic languages: Polish is suggested to be the closest relative of East Slavic languages, forming a clade with them, distinct from the rest of West Slavic languages (see Fig. 1 in Gray and Atkinson, 2003).

The picture somewhat changes in Bouckaert et al., 2012/2013, where three Slavic clades are distinguished on the tree: South, East and West, but Polish — a West Slavic language — falls within the midst of the East clade, being closer to Ukrainian-Belarusian than even Russian does (see Figs. S1–S2 in Bouckaert et al., 2012/2013).

We would like to stress, however, that pointed out hereby questionable, and indeed erroneous to our opinion lexical inferences and elements of the obtained trees in the two aforementioned publications, do not discredit the Bayesian MCMC method for language genealogical classification. We suggest that problematic elements in the branch pattern in the Slavic section in Gray & Atkinson (2003) and Bouckaert et al. (2012) trees have likely resulted due to incorrect input data.

In both studies (Gray and Atkinson 2003, Bouckaert et al. 2012/2013) authors have also attempted to date major nodes of the tree of Indo-European languages including the Balto-Slavic branch. The initial split of Proto-Slavic dates back to AD 700 according to Gray and

Atkinson 2003 and falls within the range AD 250–650 according to Bouckaert et al. 2012/2013. Bouckaert et al.'s date range fits better the available historical and archaeological evidence and does not seriously contradict our lexicostatistical date (AD 100). As for internal nodes dating in Bouckaert et al. 2012/2013, the authors provide wide and partially overlapping temporal estimates for the majority of nodes that probably require further elaboration.

The formal classification of the world's languages, proposed by the *Automated Similarity Judgment Program* (ASJP) project (Müller et al. 2013; Holman et al. 2011), is based on the non-etymologized 40-item wordlists. The average Levenshtein distances between individual wordforms with the same meaning yield the distance matrix between languages, which is further elaborated by the neighbor joining algorithm (bootstrap tests are not applied). In the current version of the ASJP tree (ver. 4, October 2013; Müller et al. 2013), the Balto-Slavic lects are united into one clade, which further splits into two clades: Baltic and Slavic thus being in agreement with the traditional reconstruction. Further classification of the Slavic language raises some questions. East Slavic *taxa* indeed form a distinct clade, but the distance between the Proto-Slavic and Proto-East Slavic nodes is relatively short, so the initial split of Proto-Slavic tends to be three-way: (1) Russian, (2) Ukrainian-Belarusian and (3) all others. Classification of the rest of Slavic lects appears to be more problematic. South Slavic *taxa* form a distinct clade, which turns out, however, to be inserted in the very midst of West Slavic languages: Czech-Slovak is suggested to be the closest relative of South Slavic languages, forming a clade with them, distinct from the rest of West Slavic languages.

It remains beyond the scope of the present paper whether such an approach as accepted in the *ASJP* project is suitable for detailed language genealogical classification, but we should note that, unfortunately, the *ASJP* input wordlists are not free from errors (*ASJP* lexical dataset v.16, see Wichmann et al. 2013). For example, there are, in our opinion, at least 3 lexicographic errors in the Russian 40-item list:

- 'dog': "py~os"; actually *pës* is an obsolete term, rarely used in the generic meaning 'dog'.
- 'hand': "ky~isy~ty~"; actually *kist'* is a specific technical term, rarely used in everyday language.
- 'road': "puty~"; actually *put'* means 'path' (normally metaphorically).

By its very definition, the *ASJP* algorithm is sensitive to transcriptional errors. In our opinion, there are at least 19 transcriptional inaccuracies in the Russian 40-item list (for the ASCII-based transcription system used in the *ASJP* project, see Brown et al. 2008):

- ‘one’: “ody~in”; the correct form should be “3dy~i4” or “3cy~i4”.
- ‘tree’: “dy~Ery~Ev3”; the correct form should be “dy~Ery~iv3”.
- ‘blood’: “krovy~”; the correct form should be “krofy~”.
- ‘horn’: “rog”; the correct form should be “rok”.
- ‘eye’: “glaz”; the correct form should be “glas”.
- ‘nose’: “nos”; the correct form should be “4os”.
- ‘tooth’: “zub”; the correct form should be “zup”.
- ‘tongue’: “yaz3k”; the correct form should be “iz3k”.
- ‘knee’: “koly~En3”; the correct form should be “koly~E43”.
- ‘breasts’: “grudy~”; the correct form should be “gruty~”.
- ‘heart’: “sy~3rtc3”; the correct form should be “sy~erc3”.
- ‘see’: “vy~idy~E”; the correct form should be “vy~idy~i”.
- ‘hear’: “sl3Sa”; the correct form should be “sl3S3”.
- ‘sun’: “sonc3”; the correct form should be “so4c3”.
- ‘star’: “zvy~Ezda”; the correct form should be “zvy~izda”.
- ‘night’: “noC”; the correct form should be “4oC”.
- ‘full’: “poln”; the correct form should be “pol4”.
- ‘new’: “nov”; the correct form should be “4ov”.
- ‘name’: “imy~a”; the correct form should be “imy~i”.

Besides, there is inconsistency in treatment of paradigmatic endings. On the one hand, substantives are quoted as whole nominative forms, e.g., *koly~En3* ‘knee’, *sy~3rtc3* ‘heart’, where the final element *-3* is the nom. sg. exponent. On the other hand, adjectives are quoted as bare stems without endings, e.g., *poln* instead of the nominative form *poln3y* (*-3y* is the nom. sg. m. exponent). Verbs occupy an intermediate position: these lack person & number endings, e.g., *vy~idy~E* ‘to see’, *sl3Sa* ‘to hear’, but retain the final vowels (*-E*, *-a*), which the represent preterite stem exponents.

Taken together, we suppose that the aforementioned inaccuracies in input dataset compilation should have influenced the resulted *ASJP* reconstruction of the Slavic tree topology.

Linguistic datasets and related files are available as two files:

S1 Dataset (zip-archive):

- bslav.dbf, bslav.var, bslav.inf, lexical dataset in STARLING format (multistate matrix with synonyms allowed). This dataset exported in MS EXCEL format is available as Table A in S3 File.
- bslav.nex, the same dataset as a binary matrix in NEXUS format.
- *.tre, some of the discussed trees in NEWICK format;
- NEXUS files for NeighborNet networks.

S3 File (MS Excel format):

- Table A in S3 File, lexical dataset (multistate matrix with synonyms allowed).
- Table B in S3 File, reverse distance matrix, generated from the multistate matrix (Table A in S3 File) in the Starling software.
- Table C in S3 File, distance matrix, generated from the binary matrix (bslav.nex) in the SplitsTree4 software.

S2: References

Atkinson QD, Gray RD (2006) How old is the Indo-European language family? Progress or more moths to the flame? In: Forster P, Renfrew C, editors. Phylogenetic Methods and the Prehistory of Languages. Cambridge: The McDonald Institute for Archaeological Research, p. 91–109.

Balanovsky O, Dibirova K, Dybo A, Mudrak O, Frolova S, Pocheshkhova E, Haber M, Platt D, Schurr T, Haak W, et al. 2011. Parallel evolution of genes and languages in the Caucasus region. Mol Biol Evol 28: 2905–2920.

Barford PM. 2001. The Early Slavs: Culture and Society in Early Medieval Eastern Europe. Ithaca: Cornell University Press.

Bernstein SB. 1961. Ocherk sravnitel’noj grammatiki slavyanskikh yazykov [Comparative Grammar of the Slavic Languages], vol. 1. Moscow: Izd-vo Akademii nauk SSSR.

Bezlaj F. 2003. Položaj slovenščine v okviru slovanskih jezikov. In: Bezlaj F. Zbrani jezikoslovni spisi, vol. 1. Ljubljana: Založba ZRC.

Blažek V. 2007. From August Schleicher to Sergei Starostin: On the development of tree-diagram models of the Indo-European languages. Journal of Indo-European studies 35: 82–109.

Bouckaert R, Lemey P, Dunn M, Greenhill SJ, Alekseyenko AV, Drummond AJ, Gray RD, Suchard MA, Atkinson QD. 2012. Mapping the Origins and Expansion of the Indo-European Language Family. Science 337: 957–960. With corrections and revised supplementary materials in: Science 342 (20 December 2013): 1446.

Brown CH, Holman EW, Wichmann S, Velupillai V. 2008. Automated classification of the world's languages: A description of the method and preliminary results. STUF — Language Typology and Universals 61.4: 285–308.

Bryant D, Moulton V. 2004. NeighborNet: an agglomerative algorithm for the construction of phylogenetic networks. Mol Biol Evol 21: 255–265.

Burlak SA, Starostin SA. 2005. Sravnitel'no-istoricheskoe yazykoznanie [Historical Linguistics], 2nd ed. Moscow: Academia.

Curta F. 2001. The Making of the Slavs: History and Archaeology of the Lower Danube Region, c. 500–700. Cambridge: Cambridge University Press.

Dyen I, Kruskal J, Black P. 1997. Comparative Indo-European Database. This file was last modified on Feb 5, 1997. http://www.wordgumbo.com/ie/cmp/ [Accessed 07.07.2014]

Gascuel O. 1997. BIONJ: an improved version of the NJ algorithm based on a simple model of sequence data. Mol Biol Evol 14: 685–695.

Goloboff PA, Farris JS, Nixon KC. 2008. TNT, a free program for phylogenetic analysis. Cladistics 24/5: 774–786.

Gray RD, Atkinson QD. 2003. Language-tree divergence times support the Anatolian theory of Indo-European origin. Nature 426: 435–439.

Heather P. 2010. Empires and Barbarians: The Fall of Rome and the Birth of Europe. Oxford: Oxford University Press.

Holman EW, Brown CH, Wichmann S, et al. 2011. Automated dating of the world's language families based on lexical similarity. Current Anthropology 52.6: 841–875.

Huelsenbeck JP, Ronquist F. 2001. MrBayes: Bayesian inference of phylogenetic trees. Bioinformatics 17/8: 754–755.

Huson DH, Bryant D. 2006. Application of phylogenetic networks in evolutionary studies. Mol Biol Evol 23/2: 254–267.

Kassian A. 2015. Towards a formal genealogical classification of the Lezgian languages (North Caucasus): testing various phylogenetic methods on lexical data. PLoS ONE 10(2): e0116950. doi:10.1371/journal.pone.0116950.

Kassian A, Starostin G, Dybo A, Chernov V. 2010. The Swadesh wordlist. An attempt at semantic specification. Journal of Language Relationship 4: 46–89.

Lekov I. 1958. Znachenieto na gramaticheskite, slovoobrazovatelni i leksikalni danni za klasifikatsiyata na slavyanskite ezitsi ot savremenno gledische. In: Slavyanskaya filologiya. IV Mezhdunarodnyj syezd slavistov 2. Moscow: Izd-vo Akademii nauk SSSR.

Makarenkov V, Kevorkov D, Legendre P. 2006. Phylogenetic Network Construction Approaches. In: Arora DK, Berka RM, Singh GB, editors. Applied Mycology and Biotechnology, vol. 6: Bioinformatics. Amsterdam: Elsevier, p. 61–98.

Müller A, Velupillai V, Wichmann S, Brown CH, Holman EW, et al. 2013 ASJP World Language Trees of Lexical Similarity: Version 4 (October 2013): http://asjp.clld.org/download [Accessed 07.05.2015]

Novotná P, Blažek V. 2007. Glottochronology and its application to the Balto-Slavic languages. Baltistica 42/2: 185–210; Baltistica 42/3: 323–346.

Saenko MN. 2013. Rekonstruktsiya praslavyanskogo spiska Svodesha [Reconstruction of the Proto-Slavic Swadesh wordlist]. Journal of Language Relationship 10: 139–148.

Saitou N, Nei M. 1987. The neighbor-joining method: A new method for reconstructing phylogenetic trees. Mol Biol Evol 4: 406–425.

Sedov VV. 1979. Proiskhozhdenie i rannyaya istoriya slavyan [Origin and early history of the Slavs]. Moscow: Nauka.

Sneath PHA, Sokal RR. 1973. Numerical Taxonomy. San Francisco: W.H. Freeman and Company.

Sobolev AN. 2000. Voprosy drevnejshej istorii yuzhnoslavyanskikh yazykov i areal'naya lingvistika. Južnoslovenski filolog 56/3–4: 1035–1050.

Starostin GS. 2010. Preliminary lexicostatistics as a basis for language classification: A new approach. Journal of Language Relationship 3: 79–116.

Starostin GS (ed.). 2011–2015. The Global Lexicostatistical Database. Moscow/Santa Fe: Center for Comparative Studies at the Russian State University for the Humanities; Santa Fe Institute. Available: http://starling.rinet.ru/new100 [Accessed 07.05.2015].

Starostin SA. 1989/2007. Sravnitel'no-istoricheskoe yazykoznanie i leksikostatistika. In: Starostin 2007: 407–447. [First publ. in: Lingvisticheskaya rekonstruktsiya i drevne-

jshaya istoriya Vostoka. Moscow, 1989, p. 3–39. Eng. version: Starostin SA 1999/2000.]

Starostin SA. 1993/2007. Rabochaya sreda dlya lingvista. In: Starostin 2007: 481–496. [First publ. in: Bazy dannykh po istorii Evrazii v srednie veka 2. Moscow: Institut vostokovedeniya RAN, 1993, p. 50–64. Republ. in: Gumanitarnye nauki i novye informatsionnye tekhnologii. Moscow: RGGU, 1994, p. 7–23.]

Starostin SA (ed.). 1998–2005. *The Tower of Babel. An Etymological Database Project*. http://starling.rinet.ru/ [Accessed 07.05.2015]

Starostin SA. 1999/2000. Comparative-historical linguistics and lexicostatistics. In: Historical Linguistics and Lexicostatistics. Melbourne: Association for the History of Language, 1999, p. 3–50 [Republ. in: Time Depth in Historical Linguistics. Oxford: McDonald Institute for Archaeological Research, 2000, p. 223–259.]

Starostin SA. 2007. Trudy po yazykoznaniyu [Works in Linguistics]. Moscow: Yazyki slavyanskikh kultur.

Starostin SA. 2007a. Opredelenie ustojchivosti bazisnoj leksiki [Defining the stability of basic lexicon]. In: Starostin 2007: 827–839.

Stieber Z. 1972. O drevnikh slovensko-zapadnoslavyanskikh svyazyakh [On ancient Slovenian-West Slavic connections]. In: Russkoe i slavyanskoe yazykoznanie. K 70-letiyu chl.-korr. AN SSSR R. I. Avanesova. Moscow: Nauka.

Sussex R, Cubberley P. 2006. The Slavic languages. Cambridge: Cambridge University Press.

Wichmann S, Müller A, Wett A, Velupillai V, et al. 2013. The ASJP Database, version 16: http://asjp.clld.org/download [Accessed 07.05.2015].
