# Supplementary material for: Genetic Heritage of the Balto-Slavic Speaking Populations: A Synthesis of Autosomal, Mitochondrial and Y-Chromosomal Data
Source: PLoS One. 2015 Sep 2;10(9):e0135820. doi: 10.1371/journal.pone.0135820 (PMC4558026; doi:10.1371/journal.pone.0135820)
Supplement: S1 Text — (DOCX) [file pone.0135820.s010.docx]

**SUPPLEMENTARY INFORMATION 1**

**S1: Datasets**

When assembling datasets for this study, we aimed to represent all Balto-Slavic populations and this goal has been largely achieved. Most linguists recognize two extant East Baltic (Lithuanian, Latvian) and 11 Slavic languages (Polish, Czech, Slovak, Sorbian, Ukrainian, Russian, Belarusian, Bulgarian, Macedonian, Slovenian and Serbo-Croatian). Populations speaking 11 of these 13 languages have national states named after them (Lithuania, Latvia, Poland, Czech Republic, Slovakia, Ukraine, Russia, Belarus, Bulgaria, Macedonia, Slovenia); populations speaking Serbo-Croatian live in four different states (Serbia, Montenegro, Croatia, and Bosnia); finally, the population speaking Sorbian lives in Germany. These 16 Balto-Slavic populations are named “*ethnic populations*” throughout the paper, whereas Kashubians were considered here as a subgroup of Poles. Each ethnic population was represented in every set of genetic data with two minor exceptions: Sorbs are missing from the mtDNA dataset and Montenegrins are missing from the mtDNA and NRY datasets. Since many ethnic populations occupy large areas, we aimed to represent each of them by a set (rather than by single) of “*local populations”*, i.e. populations of the particular provinces within the country.

*The NRY dataset* includes 67 local sampling points of Balto-Slavic populations and comprises 6,079 male samples. Among them, data for 1,254 samples are reported for the first time and 1,138 samples are updated from our previous work (Table L in S1 File). Table K in S1 File combines this study’s results with data previously published on Balto-Slavic populations with lower phylogenetic resolution. Table N in S1 File presents data on context populations of Europe used for comparative purposes.

*The mtDNA dataset* includes 49 local Balto-Slavic populations and comprises 6,876 samples, 917 are reported here for the first time (Table C in S1 File). In some analyses data from other Central and East European populations were included.

*The whole genome SNP dataset* includes 1297 worldwide individuals, 70 presented here for the first time, and 1227 from the literature (Table M in S1 File). This dataset includes in total 296 samples representing 16 Balto-Slavic populations.

The samples presented here for the first time (Tables C, K, M in S1 File) were collected in rural areas, where low population mobility diminished the contribution of recent migrations. All sampled volunteers identified their four grandparents as members of the given ethnic group and were unrelated for at least three generations. Informed consent was obtained from each participant.

**S1:** **Methods**

**Whole-genome data assembling and pre-processing**

Different sets of regional populations genotyped with various platforms were included depending on particular analysis (Table M in S1 File). The overlap of SNPs between Illumina 610K, 650K and 660K arrays was around 500K, whereas between Illumina and Affymetrix genotyping platforms was around 74K. Because the reduction of the SNP number in case of using a cross-platform dataset did not affect the resulted structure of populations in PCA and pairwise F_ST_ calculations (data not shown), the cross-platform datasets representing a Balto-Slavic genetic background at highest level were used for PCA and F_ST_ analyses. Only genotype data generated with Illumina arrays 610K, 650K and 660K were used for ADMIXTURE analysis and analysis of segments shared identical by descent.

The whole genome datasets were pre-processed with PLINK v1.07 (<http://pngu.mgh.harvard.edu/purcell/plink/>) [1] before all analyses. SNPs on the 22 autosomes with minor allele frequency >1% and missing genotype rate <3% were included in the analyses; individuals with missing genotype data >3% were excluded; relatives in the first and the second degree were detected and one individual from each pair was removed [2]. To reduce the effect of a Linkage Disequilibrium (LD), SNPs in strong LD (r^2^>0.4) were pruned out in a window of 200 nucleotides with sliding window of 25 nucleotides for datasets used in the PCA and ADMIXTURE analyses. Set of only Illumina cross-platform SNPs (around 200K shared LD-pruned SNPs between the 610K, 650K and 660K arrays) were used for ADMIXTURE [3]; set of only Illumina cross-platform SNPs (around 500K) were used in analysis of segments *identical by descent* (IBD) [4]. Sets of Illumina-Affymetrix cross-platform SNPs (around 57K of LD-pruned SNPs), encompassing higher number of Balto-Slavic populations, were used in Principal Component Analysis (PCA) [5] and pairwise F_ST_ calculations.

**Multidimensional scaling (MDS)**

MDS analysis was performed for the NRY and mtDNA datasets. Both datasets included local Balto-Slavic populations and additional European populations for context (Tables C, K, N in S1 File). Genetic distances between populations were calculated according to [6] using the DJ software (written by Y. Seryogin, and available at <http://genofond.ru>). The distances were visualized on MDS plots in Statistica 6.0 software (StatSoft. Inc., 2001).

**Principal Component Analysis (PCA)**

PCA was performed for the autosomal dataset using the *smartpca* program of the EIGENSOFT package [5] to analyze the genetic structure of Balto-Slavic-speaking populations in a European context. Populations used in PCA are listed in Table M in S1 File. The genetic structure of populations revealed by the first five principal components was examined. S1 Fig. shows PC1*vs*PC2 plot.

**ADMIXTURE**

Genomic ancestry components in Balto-Slavic speakers in the context of worldwide populations were inferred with the ADMIXTURE [3]. Populations included into the analysis are listed in Table M in S1 File. For choosing the value of K which best models the ancestry components in our dataset, we applied a cross-validation (CV) procedure following the ADMIXTURE specifications. The lowest CV error in our dataset was at K=6. Additionally, the maximum difference in Log Likelihood (LL) scores in fractions of runs with the highest LL score at each K was inspected. We assumed that a global LL maximum was achieved at a given K if 10% of the runs with the highest LL score showed minimal variation in LL scores (i.e. less than 1 LL unit). Accordingly, the global LL maximum in our dataset was presumably achieved at K2-K8, and at K12-K15. S2 Fig. shows ADMIXTURE plot for *k2*-*k20*; box-and-whisker plot of the cross validation indexes (CV) of all runs; log-likelihood scores (LLs) of 20x100 runs of the ADMIXTURE; variation in LLs in the fractions of 5%, 10% and 20% of runs that reached the highest LLs.

**Analysis of segments identical by descent**

The fast IBD (*fIBD*) algorithm [4] implemented in BEAGLE ([http://faculty.washington.edu/browning/beagle/beagle.html](http://www.sciencedirect.com/science?_ob=RedirectURL&_method=externObjLink&_locator=url&_cdi=276895&_issn=00029297&_origin=article&_zone=art_page&_plusSign=%2B&_targetURL=http%253A%252F%252Ffaculty.washington.edu%252Fbrowning%252Fbeagle%252Fbeagle.html)) was used to detect pairwise IBD segments. All populations were combined into nine groups (Table F in S1 File, S3 Fig.), where groups 1 (East and West Slavs) and 2 (South Slavs) encompassed present-day Slavic speakers, and the remaining seven groups were created based on geographic affiliations of populations (e.g. Baltic-speakers were grouped together with neighboring Estonians etc.) to represent geographic neighbors of Slavs. East and West Slavic speakers were combined together due to following reasons: most of West Slavs overlap with East Slavs in to their NRY [7] and autosomal diversity (Fig. 2a); East and West Slavic speakers occupy the same geographic niche – Central-East Europe – and are separated from South Slavs nowadays; area occupied by East and West Slavs fits better different supposed regions from where bearers of Slavic languages expanded towards west, east and south [8–10]. An IBD experiment was designed to compare the level of IBD relatedness between two groups of Slavs – the combined group of East-West Slavs and group of South Slavs (i.e. IBD relatedness within Slavs) – to the IBD relatedness between each group of Slavs – East-West Slavs and South Slavs and their respective geographic neighbors (S3 Fig., Table F in S1 File). To this end we:

a) calculated an average number of IBD segments per pair of individuals (ibd-statistic) between the group East-West Slavs (group 1) and South Slavs(group 2), i.e. IBD sharing within Slavs.

b) calculated ibd-statistics between each group of Slavs and their geographic neighbors (S3 Fig.).

c) compared ibd-statistic within Slavs with ibd-statistics between Slavs and their non-Slavic geographic neighbors.

The *fIBD* algorithm was applied to 22 autosomes in 10 iterations, and the IBD threshold was set to 1e-12. The IBD segments were divided into the following ten length classes (in centiMorgans (cM)): 0-1; 1-1.5; 1.5-2; 2-2.5; 2.5-3; 3-3.5; 3.5-4; 4-4.5; 4.5-5; >5. The average number of shared IBD segments per pair of individuals was calculated for each group combination within each length class. Considered only counts of IBD segments shared between different groups and ignored segments shared between individuals within the same group. To assess the differences in IBD sharing between group of East-West Slavs and South Slavs as compared to that shared between each groups of and their respective geographic non-Slavic neighbors we performed a statistical test as following.

Poisson regression with random effects was used to model the distribution of IBD segments shared between two individuals from two groups under comparison. Our analysis aimed to assess the differences in IBD sharing between East-West Slavs (here denoted as the **base group**) and South Slavs, compared with sharing between the **base group** and neighboring groups (Table G in S1 File).

Assume that the number of shared IBD segments of a given length interval between an individual *i* from the base group and an individual *j* from a group *k* is denoted by *x_ijk_* and has a Poisson distribution with parameters *λ_ijk_*:

log (*λ_ijk_*) *= μ + α_k_ + γ_k,i_ + τ_j_*, where

*α_k_* is a *k* group-specific effect;

exp(*μ + α_k_*) is a typical number of shared IBD segments between an individual from the base group and an individual from group *k*;

*τ_j_* is a random effect for an individual j from a comparison group, those effects are assumed to have zero expectation (*E τj* = 0) over all individuals in the comparison group;

*γ_k,i_* is a random effect for individual *i* from a base group, those effects are assumed to have zero expectation (*E γk,i* = 0) over all individuals in the base group (for all comparison groups).

The model was applied for each length interval of IBD segments separately. Under H_0_ we assume that the number of IBD segments shared between the base group and South Slavs (1) and between the base group and any respective neighboring group is

H_0_: *μ + α_1_*=*μ + α_k_*

95% confidence intervals (CI) were calculated for each comparison *base group* - *k*-*group* for all 10 classes of IBD segments length.

Similar procedure was performed to assess differences for ibd-statistic within Slavs (South Slavs *vs* East-West Slavs) and ibd-statistics for South Slavs and their respective neighboring groups (S3 Fig.).

The mean length of the IBD segments (in cM) after *N* generations since the common ancestor for a pair of individuals, assuming independent rate of recombination (recombination event can occur in any point of the genome independently), will be 1/2*N*, and lengths will be distributed exponentially [4]. Assume that migrants from central-eastern Europe have reached the Balkan region not later than the 7th century AD [9], then the mean length of an IBD segment for a pair of individuals will be around 2 cM (one generation is equal to 25-30 years). Given that most individuals in populations are connected to each other through *multiple path* *genealogies*, the length of IBD segments is likely to be longer [11,12]. Thus, in theory we may expect an excess of IBD segments around 2 cM among populations that experienced recent admixture through substantial migration.

**Analysis of molecular variation (AMOVA)**

AMOVA (implemented in the Arlequin 3.11) was applied to estimate genetic differentiation when Balto-Slavic populations were grouped according to the linguistic classification of the Balto-Slavic languages (Fig. 1, S5 Fig.). We estimated genetic variation at three hierarchical levels: (i) variation among local populations speaking the same language, (ii) variation between ethnic (defined by language) populations belonging to the same linguistic branch and (iii) variation between branches of Balto-Slavic languages (see details in Table H in S1 File). The scheme in S5 Fig. illustrates which three branching levels were considered in the analysis. Note, that when languages were subdivided into dialects, it accounted for additional portion of total variation (Table H in S1 File).

**Mantel tests**

Mantel tests were performed in Arlequin 3.11[13] to calculate the coefficients of the pairwise and partial correlations between matrices of genetic, linguistic and geographic distances. We included only those Balto-Slavic populations for which data on all three genetic systems and lexicostatistics were available. Thereby, Sorbs were excluded because of absence of mtDNA data; and Bosnians, Croatians, Serbians and Montenegrins were combined as they speak the same Serbo-Croatian language. North Russians were not included because of their significant genetic peculiarities [7,14,15]. Five distance matrices between 12 Balto-Slavic populations were created for this analysis (Table Ia-e in S1 File). Geographic distances (Table Ia in S1 File) were calculated from geographic coordinates using a spherical formula. Pairwise linguistic distances were obtained as complement-on-one of lexicostatistical similarities (Table Ib in S1 File). There were three genetic distance matrices – for autosomal, NRY and mtDNA data. Distances based on NRY (Table Id in S1 File) and mtDNA data (Table Ie in S1 File) were calculated according to [6] from the NRY (Table K in S1 File) and mtDNA (Table C in S1 File) datasets, respectively, after pooling populations speaking the same language. Mean pairwise F_ST_ values [16] were calculated for autosomal data using an in-house R-script as in [17] and PLINK v1.07 [1] based on 74K Illumina-Affymetrix cross-platform ‘post quality controlled’ SNPs (Table Ic in S1 File). Pairwise and partial correlations between every genetic matrix with the geographic and linguistic ones were used for revealing relative importance of geography and language for shaping genetic structure (Table J in S1 File); pairwise correlations among all five matrices were used for comparing variation of the three genetic systems, geography and linguistics (Fig. 5).

**S1: References**

1. Purcell S, Neale B, Todd-Brown K, Thomas L, Ferreira MAR, Bender D, et al. PLINK: a tool set for whole-genome association and population-based linkage analyses. Am J Hum Genet. 2007;81: 559–575. doi:10.1086/519795

2. Manichaikul A, Mychaleckyj JC, Rich SS, Daly K, Sale M, Chen W-M. Robust relationship inference in genome-wide association studies. Bioinformatics. 2010;26: 2867–2873. doi:10.1093/bioinformatics/btq559

3. Alexander DH, Novembre J, Lange K. Fast model-based estimation of ancestry in unrelated individuals. Genome Res. 2009;19: 1655–1664. doi:10.1101/gr.094052.109

4. Browning BL, Browning SR. A fast, powerful method for detecting identity by descent. Am J Hum Genet. 2011;88: 173–182. doi:10.1016/j.ajhg.2011.01.010

5. Patterson N, Price AL, Reich D. Population structure and eigenanalysis. PLoS Genet. 2006;2: e190. doi:10.1371/journal.pgen.0020190

6. Nei M. Genetic distance between populations. Am Nat. 1972;106: 283–92.

7. Balanovsky O, Rootsi S, Pshenichnov A, Kivisild T, Churnosov M, Evseeva I, et al. Two sources of the Russian patrilineal heritage in their Eurasian context. Am J Hum Genet. 2008;82: 236–250. doi:10.1016/j.ajhg.2007.09.019

8. Sedov VV. Slaviane: Istoriko-arheologicheskoe issledovanie [Slavs: Historical and archaeological study]. Moskva: Yazyki slavianskoi kultury; 2002.

9. Curta F. The Making of the Slavs: History and Archaeology of the Lower Danube Region. Cambridge University Press; 2001.

10. Toporov VN, Trubachev ON. Lingvisticheskij analiz gidronimov Verkhnego Podneprov’ya [Linguistic study of hydronyms of Upper Dnieper]. Moskva: Akademiya Nauk SSSR; 1962.

11. Browning SR, Browning BL. Identity by descent between distant relatives: detection and applications. Annu Rev Genet. 2012;46: 617–633. doi:10.1146/annurev-genet-110711-155534

12. Ralph P, Coop G. The Geography of Recent Genetic Ancestry across Europe. PLoS Biol. 2013;11: e1001555. doi:10.1371/journal.pbio.1001555

13. Excoffier L, Laval G, Schneider S. Arlequin (version 3.0): an integrated software package for population genetics data analysis. Evol Bioinforma Online. 2005;1: 47–50.

14. Morozova I, Evsyukov A, Kon’kov A, Grosheva A, Zhukova O, Rychkov S. Russian ethnic history inferred from mitochondrial DNA diversity. Am J Phys Anthropol. 2012;147: 341–351. doi:10.1002/ajpa.21649

15. Khrunin AV, Khokhrin DV, Filippova IN, Esko T, Nelis M, Bebyakova NA, et al. A genome-wide analysis of populations from European Russia reveals a new pole of genetic diversity in northern Europe. PloS One. 2013;8: e58552. doi:10.1371/journal.pone.0058552

16. Weir BS, Cockerham CC. Estimating F-Statistics for the Analysis of Population Structure. Evolution. 1984;38: 1358. doi:10.2307/2408641

17. Gallego Romero I, Basu Mallick C, Liebert A, Crivellaro F, Chaubey G, Itan Y, et al. Herders of Indian and European cattle share their predominant allele for lactase persistence. Mol Biol Evol. 2012;29: 249–260. doi:10.1093/molbev/msr190

**S1 File: Figures**

S1 Fig. PC1*vs*PC2 plot based on whole genome SNP data (PC1=0.53; PC2=0.34).

S2 Fig. ADMIXTURE plot (k2-k20) (A). Box and whiskers plot of the cross validation (CV) indexes of all runs of the ADMIXTURE analysis (B). Log-likelihood (LL) scores of all runs (C). Variation in LL scores in the fractions (5%, 10%, 20% shown in dark green, middle green and light green, respectively) of runs that reached the highest LLs) (D).

S3 Fig. Schematic representation of groups of populations used in the IBD analysis. Populations within each group are listed in Table F in S1 File. Source of the Europe contour map: <http://www.conceptdraw.com/How-To-Guide/geo-map-europe>.

S4 Fig. Distribution of the average number of IBD segments between group of East-West Slavs and their geographic neighbors. Russians from Northern regions are considered separately from the group of north-east Europeans. The x-axis indicates ten classes of IBD segment length (in cM); the y-axis indicates the average number of shared IBD segments per pair of individuals within each length class.

S5 Fig. Hierarchical levels of genetic variation used in AMOVA.

**S1 File: Tables**

Table A in S1 File. Matrix of pairwise Nei distances (D_Nei_) between Balto-Slavic populations based on Y-chromosome data.

Table B in S1 File. Matrix of mean population pairwise F_ST_ for Balto-Slavic populations calculated from autosomal SNP data.

Table C in S1 File. Frequencies of the mtDNA haplogroups in Balto-Slavic and some other European populations.

Table D in S1 File. Matrix of pairwise Nei distances (D_Nei_) between Balto-Slavic populations based on mtDNA data.

Table E in S1 File. Predicting the country affiliation for 53 Balto-Slavic populations from their Y-chromosomal composition.

Table F in S1 File. Groups of populations used in IBD analysis.

Table G in S1 File. Summary statisctics of IBD analysis.

Table H in S1 File. Analysis of molecular variance (AMOVA) in Balto-Slavic populations.

Table I in S1 File. Matrices of geographic (a), lexicostatistical (b) and genetic (c,d,e) distances between Balto-Slavic populations used in Mantel Tests.

Table J in S1 File. Results for Mantel tests on genetic, lexicostatistical and geographic distances.

Table K in S1 File. Frequencies of the NRY haplogroups in Balto-Slavic populations.

Table L in S1 File. Frequencies of NRY haplogroups in 29 Balto-Slavic populations presented here for the first time.

Table M in S1 File. Populations used in whole-genome SNP analyses.

Table N in S1 File. Frequencies of the NRY haplogroups in non-Balto-Slavic populations of Europe.

**S1 File: Online resourses**

**Data access**

The whole genome SNP data generated in this study are available in the National Center for Biotechnology Information – Gene Expression Omnibus (NCBI GEO accession number GSE71049) as well as in PLINK format in our website at [www.ebc.ee/free_data](http://www.ebc.ee/free_data). The NRY dataset is presented in Table N in S1 File; mtDNA HVS1 sequences are available in the National Center for Biotechnology Information (GenBank accession numbers KT261802 - KT262718).

**Web resources**

The Global Lexicostatistical Database, <http://starling.rinet.ru/new100/>

BEAGLE, [http://faculty.washington.edu/browning/beagle/beagle.html](http://www.sciencedirect.com/science?_ob=RedirectURL&_method=externObjLink&_locator=url&_cdi=276895&_issn=00029297&_origin=article&_zone=art_page&_plusSign=%2B&_targetURL=http%253A%252F%252Ffaculty.washington.edu%252Fbrowning%252Fbeagle%252Fbeagle.html)

PLINK v1.07 software, <http://pngu.mgh.harvard.edu/purcell/plink/>
