# Supplementary material for: Genetic Heritage of the Balto-Slavic Speaking Populations: A Synthesis of Autosomal, Mitochondrial and Y-Chromosomal Data
Source: PLoS One. 2015 Sep 2;10(9):e0135820. doi: 10.1371/journal.pone.0135820 (PMC4558026; doi:10.1371/journal.pone.0135820)
Supplement: S2 Fig — (PDF) [file pone.0135820.s003.pdf]

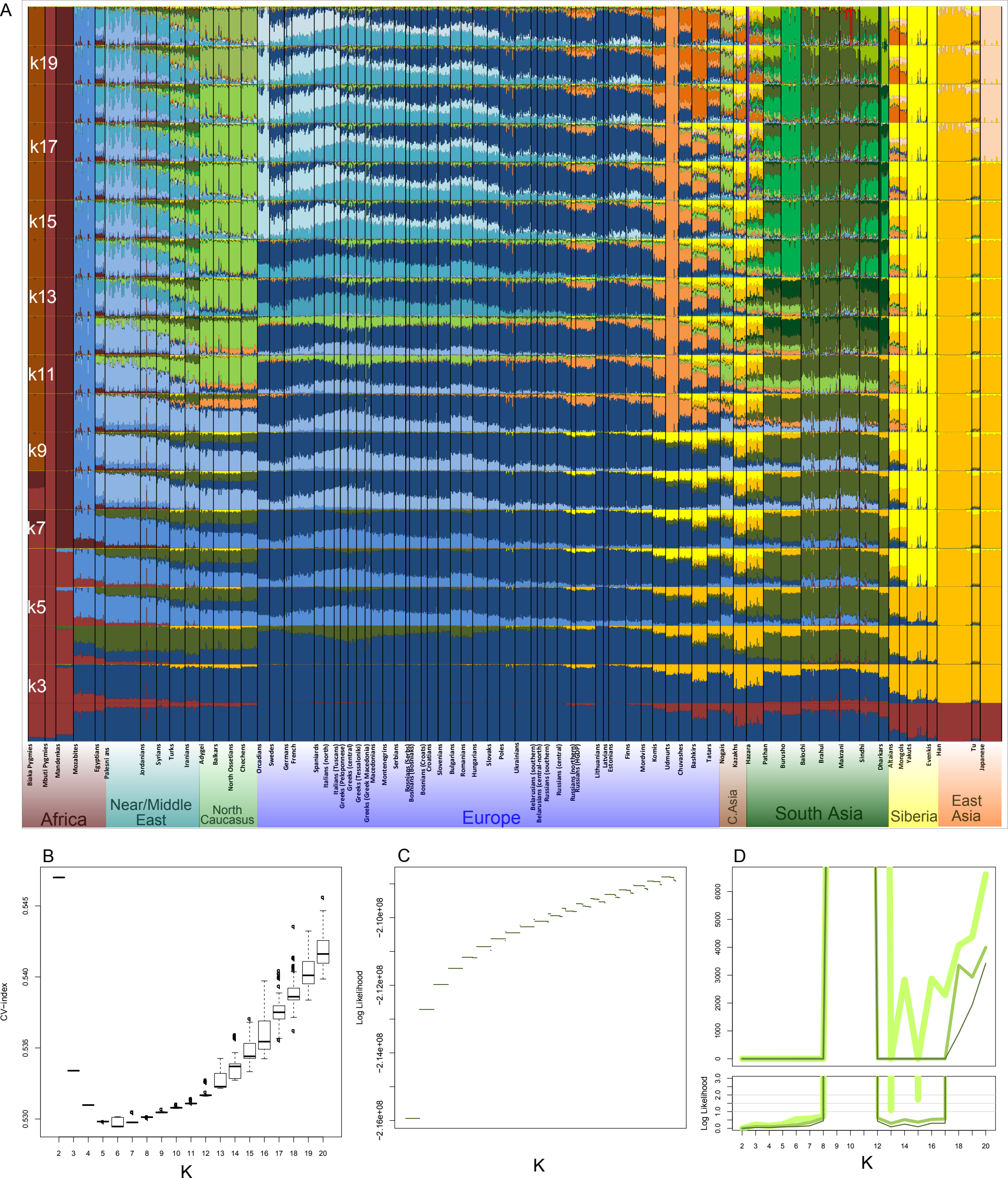
A
k19
k17
k15
k13
k11
k9
k7
k5
k3
Biaka Pygmies
Mbuti Pygmies
Mandenkas
Mozabites
Egyptians
Palesni ans
Jordanians
Syrians
Turks
Iranians
Adygei
Balkars
North Ossetians
Chechens
Orcadians
Swedes
Germans
French
Spaniards
Italians (north)
Italians (Tuscans)
Greeks (Peloponnese)
Greeks (central)
Greeks (Tessaloniki)
Greeks (Greek Macedonia)
Macedonians
Montenegrins
Serbians
Bosnians (Serbs)
Bosnians (Bosniaks)
Bosnians (Croats)
Croatians
Slovenians
Bulgarians
Romanians
Hungarians
Slovaks
Poles
Ukrainians
Belarusians (southern)
Belarusians (central-north)
Russians (southern)
Russians (central)
Russians (northern)
Russians (HGDP)
Lithuanians
Latvians
Estonians
Finns
Mordvins
Komis
Udmurts
Chuvashes
Bashkirs
Tatars
Nogais
Kazakhs
Hazara
Pathan
Burusho
Balochi
Brahui
Makrani
Sindhi
Dharkars
Altaians
Mongols
Yakuts
Evenkis
Han
Tu
Japanese
Africa
Near/Middle East
North Caucasus
Europe
C.Asia
South Asia
Siberia
East Asia
B
CV-index
0.530
0.535
0.540
0.545
K
C
Log Likelihood
−2.10e+08
−2.12e+08
−2.14e+08
−2.16e+08
K
D
0
1000
2000
3000
4000
5000
6000
Log Likelihood
0.0
1.0
2.0
3.0
K
